# On Orlicz classes defined in terms of associated weight functions

**DOI:** 10.1007/s00605-024-01991-x

**Published:** 2024-05-28

**Authors:** Gerhard Schindl

**Affiliations:** Fakultät für Mathematik, Universität Wien, Oskar-Morgenstern-Platz 1, 1090 Vienna, Austria

**Keywords:** Orlicz classes and Orlicz spaces, N-functions, Associated weight functions, Weight sequences, Dual sequence, 26A12, 26A48, 26A51, 46E30

## Abstract

N-functions and their growth and regularity properties are crucial in order to introduce and study Orlicz classes and Orlicz spaces. We consider N-functions which are given in terms of so-called associated weight functions. These functions are frequently appearing in the theory of ultradifferentiable function classes and in this setting additional information is available since associated weight functions are defined in terms of a given weight sequence. We express and characterize several known properties for N-functions purely in terms of weight sequences which allows to construct (counter-) examples. Moreover, we study how for abstractly given N-functions this framework becomes meaningful and finally we establish a connection between the complementary N-function and the recently introduced notion of the so-called dual sequence.

## Introduction

Let us start by recalling briefly the basic definitions of Orlicz classes and Orlicz spaces, we refer to [11, 17] and to the informative summary presented in [1]. For this let *F* be a so-called *N-function*, see Definition 3.1 below and [11, Chapter 1, §1, 3, p. 6], [17, Sect. 2.1, p. 13], [1, Def. 2.1]. Moreover, let $\Omega$ be a bounded and closed set in $\mathbb {R}^d$ and consider on $\Omega$ the usual Lebesgue measure. Then the *Orlicz class* is given by

$$\begin{aligned} \mathcal {L}_F(\Omega ):=\left\{ u:\Omega \rightarrow \mathbb {R},\;\text {measurable}:\;\;\;\int _{\Omega }F(u(x))dx<+\infty \right\} , \end{aligned}$$

and the *Orlicz space* by

$$\begin{aligned} \mathcal {L}^{*}_F(\Omega ):=\left\{ u:\Omega \rightarrow \mathbb {R},\;\text {measurable}: \int _{\Omega }u(x)v(x)dx<+\infty ,\;\;\;\forall \;v\in \mathcal {L}_{F^c}(\Omega )\right\} , \end{aligned}$$

with $F^c$ denoting the so-called *complementary N-function* which is again an N-function, see Sect. 6.1 and [11, Chapter 1, §2, p. 11], [17, Sect. 1.3, p. 6], [1, Def. 2.2]. If we do not need to specify the set $\Omega$ we omit it and only write $\mathcal {L}_F$ resp. $\mathcal {L}^{*}_F$. In [17, Chapter I, Thm. 2, Cor. 3], by revisiting a result by de la Vallée Poussin, it has been shown how such a growth restriction expressed in terms of certain convex functions *F* is arising naturally. In the literature the aforementioned sets are occasionally also defined in an even more general measure theoretic setting and slightly different assumptions on *F* are used; e.g. using *Young functions* in [17], see Remark 3.15 for more details.

In order to study these classes several growth and regularity assumptions for *F* and $F^c$ are considered frequently in the literature. Most prominent are the so-called $\Delta _2$, $\Delta _3$, $\Delta ^2$ and $\Delta '$ condition for *F*, see e.g. [11, Chapter I, §4–§6], [17, Chapter II], [1, Sect. 2.2] and Sect. 7 in this work. If $F^c$ satisfies a “$\Delta$-type” property then by convention usually one writes that *F* has the corresponding “$\nabla$-type” condition (and vice versa).

The aim of this paper is to introduce and study N-functions $F_M$ which are given in terms of a given sequence $M\in \mathbb {R}_{>0}^{\mathbb {N}}$, see Definition 3.14, via the so-called *associated weight function* $\omega _M$ (see Sect. 2.2). Here the sequence is expressing the growth of $F_M$ and *M* is assumed to satisfy mild standard and growth assumptions, see Sect. 2.1. Recall that $\omega _M$ is appearing frequently in the theory of classes of ultradifferentiable (and ultraholomorphic) functions defined in terms of weight sequences and it serves also as an example for an abstractly given weight function $\omega$ in the sense of Braun–Meise–Taylor, see [2].

Consequently, $F_M$ contains additional information expressed in the underlying sequence *M* and the idea is to exploit this fact, to “combine” the ultradifferentiable-type and the Orlicz-type setting and to treat the following questions/problems:

- Study for $F_M$ the aforementioned known and important growth properties for abstractly given N-functions in terms of *M*. When given two N-functions $F_M$, $F_L$ expressed in terms of sequences *M* and *L*, then study the crucial relation between N-functions (see (3.4)) in terms of a growth comparison between *M* and *L*.
- Use this knowledge in order to construct (counter-)examples illustrating the relations and connections between the different growth conditions for N-functions.
- Compare the (partially) new growth properties for weight sequences with known conditions appearing in the ultradifferentiable setting.
- Check if these properties and conditions can be transferred from given *M*, *L* to related constructed sequences, e.g. the point-wise product $M\cdot L$ and the convolution product $M\star L$ (see (2.1)).
- Let *G* be an abstractly given N-function. Is it then possible to associate with *G* a weight sequence, say $M^G$, and to apply the derived results in order to get information for *G* itself (via using $F_{M^G}$)?
- When given *M* and $F_M$ study and establish the connection between the notions of the complementary N-function $F_M^c$ and the dual sequence *D* (w.r.t. *M*) which has been introduced in [5, Def. 2.1.40, p. 81]. This question has served as the main motivation for writing this article. The relevance of *D* is given by the fact that the so-called orders and *Matuszewska indices* for *M* and *D* are “reflected/inverted” as it has been shown in the main result [5, Thm. 2.1.43]. Concerning these notions we refer to [5, Sect. 2.1.2], [7] and the citations there for more details and precise definitions.

However, it turns out that in the weight sequence setting we cannot expect that the relevant function $t\mapsto \varphi _{\omega _M}(t):=\omega _M(e^t)$ (see (3.14)) directly is an N-function, see Remark 3.7 for more explanations. One can overcome this technical problem by using the fact that $\varphi _{\omega _M}$ is the so-called *principal part* of an N-function $F_M$, see Definition 3.8 and Corollary 3.12. On the other hand we mention that $\varphi _{\omega _M}$ also allows to compare different used notions for being a weight in the “Orlicz-setting”, see Remark 3.15 for more details.

Note that the crucial conditions for *M* in order to ensure the desired growth properties for $F_M$ are partially (slightly) different compared with the known ones used in the ultradifferentiable setting. This is mainly due to the fact that the relevant function under consideration is given by $\varphi _{\omega _M}$ and not by $\omega _M$ directly. For example, the prominent $\Delta _2$-property for N-functions (see Sect. 7.1) is also appearing as a known growth condition in the ultradifferentiable weight function setting (abbreviated by $(\omega _1)$ in this work) but the crucial condition for *M* is different (see Theorem 7.2 and the comments there).

The paper is structured as follows: In Sect. 2 all relevant definitions concerning weight sequences and (associated) weight functions are given and in Sect. 3 we recall and introduce the notions of (associated) N-functions. In Sect. 4 we focus on the study of the comparison between associated N-functions, see Theorems 4.1, 4.2, 4.4 and Corollary 4.5, and give in Sect. 4.2 several sufficient conditions on the sequences to ensure equivalence between the associated N-functions.

Section 5 is dedicated to the study of the meaning of the associated weight sequence $M^G$ when *G* is an abstractly given N-function, see Theorems 5.2 and 5.5. In Sect. 6 we introduce and study the complementary N-function $F^c_M$ (see Theorem 6.4 and Corollary 6.5) and establish the connection between $F^c_M$ and the dual sequence *D*, see the main statement Theorem 6.9. Finally, in Sect. 7 we provide a detailed study of growth and regularity conditions for N-functions in the weight sequence setting, see Theorems 7.2, 7.7, 7.12, 7.18 and Proposition 7.25. Some (counter-)examples and their consequences are mentioned as well, see (7.17) and Corollary 7.20.

### Statements and declarations

The author confirms that there are no conflicts of interests and no problems with ethical standards. All the results and ideas contained in this publication have been obtained and developed by the author.

## Weights and conditions

### Weight sequences

We write $\mathbb {N}=\{0,1,2,\dots \}$ and $\mathbb {N}_{>0}:=\{1,2,\dots \}$ and $\mathbb {R}_{>0}$ denotes the set $(0,+\infty )$ of positive real numbers.

Let a sequence $M=(M_j)_j$ of positive real numbers be given, i.e. $M\in \mathbb {R}_{>0}^{\mathbb {N}}$. We also use the corresponding sequence $\mu =(\mu _j)_j$ defined by $\mu _j:=\frac{M_j}{M_{j-1}}$, $\mu _0:=1$, and analogously for all other appearing sequences. *M* is called *normalized* if $1=M_0\le M_1$ holds. For any $\ell >0$ we put $M^{\ell }:=(M^{\ell }_j)_{j\in \mathbb {N}}$, i.e. the $\ell$-th power, and write $M\cdot L=(M_jL_j)_{j\in \mathbb {N}}$. Finally, let us introduce the *convolved sequence* $M\star L$ by

2.1 $$\begin{aligned} M\star L_j:=\min _{0\le k\le j}M_kL_{j-k},\;\,\;j\in \mathbb {N}, \end{aligned}$$

see [10, (3.15)].

*M* is called *log-convex* if

$$\begin{aligned} \forall \;j\in \mathbb {N}_{>0}:\;M_j^2\le M_{j-1} M_{j+1}, \end{aligned}$$

equivalently if $(\mu _j)_j$ is non-decreasing. If *M* is log-convex and normalized, then both $j\mapsto M_j$ and $j\mapsto (M_j)^{1/j}$ are non-decreasing and $(M_j)^{1/j}\le \mu _j$ for all $j\in \mathbb {N}_{>0}$.

*M* (with $M_0=1$) has condition *moderate growth*, denoted by $(\text {mg})$, if

$$\begin{aligned} \exists \;C\ge 1\;\forall \;j,k\in \mathbb {N}:\;M_{j+k}\le C^{j+k} M_j M_k. \end{aligned}$$

In [10] this is denoted by (*M*.2) and also known under the name *stability under ultradifferential operators.*

For our purpose it is convenient to consider the following set of sequences

$$\begin{aligned} {\mathcal{L}\mathcal{C}}:=\left\{ M\in \mathbb {R}_{>0}^{\mathbb {N}}:\;M\;\text {is normalized, log-convex},\;\lim _{j\rightarrow +\infty }(M_j)^{1/j}=+\infty \right\} . \end{aligned}$$

We see that $M\in {\mathcal{L}\mathcal{C}}$ if and only if $1=\mu _0\le \mu _1\le \dots$, $\lim _{j\rightarrow +\infty }\mu _j=+\infty$ (see e.g. [15, p. 104]) and there is a one-to-one correspondence between *M* and $\mu =(\mu _j)_j$ by taking $M_j:=\prod _{k=0}^j\mu _k$. If $M,L\in {\mathcal{L}\mathcal{C}}$, then $M\cdot L, M\star L\in {\mathcal{L}\mathcal{C}}$ (for the convolution see [10, Lemma 3.5]).

Let $M,L\in \mathbb {R}_{>0}^{\mathbb {N}}$ be given, then write $M\le L$ if $M_j\le L_j$ for all $j\in \mathbb {N}$ and $M{\preceq }L$ if $\sup _{j\in \mathbb {N}_{>0}}\left( \frac{M_j}{L_j}\right) ^{1/j}<+\infty$. Sequences *M* and *L* are called *equivalent*, denoted by $M{\approx }L$, if $M{\preceq }L$ and $L{\preceq }M$.

#### Example 2.1

Frequently we will consider the following important examples belonging to the class $\mathcal{L}\mathcal{C}$:

(i) The Gevrey-sequences $G^s$, $s>0$, given by $G^s_j:=j!^s$.
(ii) The sequences $M^{q,n}$, $q,n>1$, given by $M^{q,n}_j:=q^{j^n}$. If $n=2$, then $M^{q,2}$ is the so-called *q*-Gevrey-sequence.

### Associated weight function

Let $M\in \mathbb {R}_{>0}^{\mathbb {N}}$ (with $M_0=1$), then the *associated function* $\omega _M: \mathbb {R}\rightarrow \mathbb {R}\cup \{+\infty \}$ is defined by

$$\begin{aligned} \omega _M(t):=\sup _{j\in \mathbb {N}}\log \left( \frac{|t|^j}{M_j}\right) \;\;\;\text {for}\;t\in \mathbb {R},\;t\ne 0,\hspace{30pt}\omega _M(0):=0. \end{aligned}$$

For an abstract introduction of the associated function we refer to [12, Chapitre I], see also [10, Definition 3.1]. Note that $\omega _M$ is here extended to whole $\mathbb {R}$ in a symmetric (even) way.

If $\liminf _{j\rightarrow +\infty }(M_j)^{1/j}>0$, then $\omega _M(t)=0$ for sufficiently small *t*, since $\log \left( \frac{t^j}{M_j}\right)<0\Leftrightarrow t<(M_j)^{1/j}$ holds for all $j\in \mathbb {N}_{>0}$. (In particular, if $M_j\ge 1$ for all $j\in \mathbb {N}$, then $\omega _M$ is vanishing on [0, 1]). Moreover, under this assumption $t\mapsto \omega _M(t)$ is a continuous non-decreasing function, which is convex in the variable $\log (t)$ and tends faster to infinity than any $\log (t^j)$, $j\ge 1$, as $t\rightarrow +\infty$. $\lim _{j\rightarrow +\infty }(M_j)^{1/j}=+\infty$ implies that $\omega _M(t)<+\infty$ for each finite *t* which shall be considered as a basic assumption for defining $\omega _M$.

For given $M\in {\mathcal{L}\mathcal{C}}$ we define the *counting function* $\Sigma _M:[0,+\infty )\rightarrow \mathbb {N}$ by

2.2 $$\begin{aligned} \Sigma _M(t):=|\{j\in \mathbb {N}_{>0}:\;\;\;\mu _j\le t\}|, \end{aligned}$$

i.e. $\Sigma _M(t)$ is the maximal positive integer *j* such that $\mu _j\le t$ (and $\Sigma _M(t)=0$ for $0\le t<\mu _1$). It is known that $\omega _M$ and $\Sigma _M$ are related by the following integral representation formula, see e.g. [12, 1.8. III] and [10, (3.11)]:

2.3 $$\begin{aligned} \omega _M(t)=\int _0^t\frac{\Sigma _M(u)}{u}du=\int _{\mu _1}^t\frac{\Sigma _M(u)}{u}du. \end{aligned}$$

Consequently, $\omega _M$ vanishes on $[0,\mu _1]$, in particular on the unit interval.

By definition of $\omega _M$ the following formula is immediate:

2.4 $$\begin{aligned} \forall \;\ell >0\;\forall \;t\ge 0:\;\;\;\ell \omega _M(t^{1/\ell })=\omega _{M^{\ell }}(t). \end{aligned}$$

In [10, Lemma 3.5] for given $M,L\in {\mathcal{L}\mathcal{C}}$ it is shown that

$$\begin{aligned} \forall \;t\ge 0:\;\;\;\Sigma _{M\star L}(t)=\Sigma _M(t)+\Sigma _L(t), \end{aligned}$$

which implies by (2.3)

$$\begin{aligned} \forall \;t\ge 0:\;\;\;\omega _{M\star L}(t)=\omega _M(t)+\omega _L(t). \end{aligned}$$

Finally, if $M\in {\mathcal{L}\mathcal{C}}$, then we can compute *M* by involving $\omega _M$ as follows, see [12, Chapitre I, 1.4, 1.8] and also [10, Prop. 3.2]:

2.5 $$\begin{aligned} M_j=\sup _{t\ge 0}\frac{t^j}{\exp (\omega _{M}(t))},\;\;\;j\in \mathbb {N}. \end{aligned}$$

#### Remark 2.2

Let $M\in {\mathcal{L}\mathcal{C}}$ be given, we comment on the surjectivity of $\Sigma _M$.

- Obviously $\Sigma _M(t)\in \mathbb {N}$ for all $t\ge 0$ and $\Sigma _M$ is surjective if and only if $\mu _j<\mu _{j+1}$ for all $j\in \mathbb {N}_{>0}$, i.e. if $j\mapsto \mu _j$ is strictly increasing: In this case we have $\Sigma _M(t)=j$ for all $\mu _j\le t<\mu _{j+1}$, $j\in \mathbb {N}_{>0}$, and $\Sigma _M(t)=0$ for $t\in [0,\mu _1)$.
- Note that $\mu _j<\mu _{j+1}$ for all *j* does not hold automatically for all sequences belonging to the set $\mathcal{L}\mathcal{C}$. However, when given $M\in {\mathcal{L}\mathcal{C}}$, then we can always find $\widetilde{M}\in {\mathcal{L}\mathcal{C}}$ such that *M* and $\widetilde{M}$ are equivalent and such that the corresponding sequence of quotients $(\widetilde{\mu }_j)_{j\ge 1}$ is strictly increasing, see [6, Lemma 3.18]. This formal switch allows to avoid technical complications resp. to simplify arguments. More precisely, in [6, Lemma 3.18] it has been shown that even
  2.6 $$\begin{aligned} 0<\inf _{j\in \mathbb {N}}\frac{\mu _j}{\widetilde{\mu }_j}\le \sup _{j\in \mathbb {N}}\frac{\mu _j}{\widetilde{\mu }_j}<+\infty , \end{aligned}$$
  which clearly implies $M \approx \widetilde{M}$. We write $M \cong N$ if (2.6) holds for the corresponding sequences of quotients $\mu$, $\nu$.

### Growth properties for abstractly given weight functions

Let $\omega :[0,+\infty )\rightarrow [0,+\infty )$, we introduce the following growth and regularity conditions

$$\begin{aligned}{} & {} {(\omega _1)}:\;\;\;\omega (2t)=O(\omega (t))\hspace{15pt}t\rightarrow +\infty ,\\{} & {} {(\omega _3)}:\;\;\;\log (t)=o(\omega (t))\hspace{15pt}t\rightarrow +\infty ,\\{} & {} {(\omega _4)}:\;\;\;\varphi _{\omega }:t\mapsto \omega (e^t)\;\text {is a convex function on}\;\mathbb {R}. \end{aligned}$$

These conditions are named after [18]. $(\omega _1)$, $(\omega _3)$ and $(\omega _4)$ are standard assumptions in the theory of ultradifferentiable functions defined by so-called *Braun–Meise–Taylor weight functions* $\omega$, see [2].

We write that $\omega$ has ${(\omega _0)}$ if $\omega$ is continuous, non-decreasing, $\omega (t)=0$ for all $t\in [0,1]$ (normalization) and $\lim _{t\rightarrow +\infty }\omega (t)=+\infty$. Finally let us put

$$\begin{aligned} {\mathcal {W}_0}:=\{\omega :[0,\infty )\rightarrow [0,\infty ): \omega \;\text {has}\;{(\omega _0)},{(\omega _3)},{(\omega _4)}\}. \end{aligned}$$

If $M\in {\mathcal{L}\mathcal{C}}$ then $\omega _M\in {\mathcal {W}_0}$, see e.g. [9, Lemma 3.1] and the citations there.

## N-functions in the weight sequence setting

### Basic definitions and abstractly given N-functions

We revisit the basic definitions from [11, Chapter I, §1, 3, p. 6] and [1, Def. 2.1]. Consider $f:[0,+\infty )\rightarrow [0,+\infty )$ with the following properties:

(I) *f* is right-continuous and non-decreasing;
(II) $f(0)=0$ and $f(t)>0$ for all $t>0$;
(III) $$\lim _{t\rightarrow +\infty }f(t)=+\infty $$.

Then we give the following definition, see e.g. [11, Chapter I, §1, 3, p. 6].

#### Definition 3.1

Let $f:[0,+\infty )\rightarrow [0,+\infty )$ be having (I), (II) and (III). The function $F:\mathbb {R}\rightarrow [0,+\infty )$ defined by

3.1 $$\begin{aligned} F(x):=\int _0^{|x|}f(t)dt, \end{aligned}$$

is called an *N-function.*

#### Remark 3.2

In [17, Sect. 1.3, Thm. 1, Cor. 2; Sect. 2.1] an analogous integral representation for N-resp. even Young functions has been obtained with the integrand *f* (“density”) being non-decreasing and *left-continuous*. Thus the authors are working with the left-derivative of *F*. Since we are focusing on the weight sequence case, see (3.14) in Sect. 3.2, we have to involve the counting function $\Sigma _M$ from (2.2) which is right-continuous by the very definition and so we prefer to work within the above setting.

Every N-function *F* satisfies the following properties, see [11, Chapter I, §1, 4, p. 7]:

- $F(0)=0$ (normalization) and $F(x)>0$ for all $x\ne 0$,
- *F* is even, non-decreasing, continuous, and convex.
- The convexity and $F(0)=0$ imply that
  3.2 $$\begin{aligned} \forall \;0\le t\le 1\;\forall \;u\ge 0:\;\;\;F(tu)\le tF(u), \end{aligned}$$
  see [11, (1.14)]. This holds since by convexity we have $F(tx+(1-t)y)\le tF(x)+(1-t)F(y)$ for all $0\le t\le 1$ and $x,y\ge 0$ and then set $y=0$.
- Finally, let us recall
  3.3 $$\begin{aligned} \lim _{t\rightarrow 0}\frac{F(t)}{t}=0,\hspace{20pt}\lim _{t\rightarrow +\infty }\frac{F(t)}{t}=+\infty , \end{aligned}$$
  see [11, (1.15), (1.16)], and which follows from (II) resp. (III) for *f*.

When given two N-functions (or even arbitrary functions) $F_1,F_2: [0,+\infty )\rightarrow [0,+\infty )$, then write $F_1{\preceq _{\mathfrak {c}}}F_2$ if

3.4 $$\begin{aligned} \exists \;K>0\;\exists \;t_0>0\;\forall \;t\ge t_0:\;\;\;F_1(t)\le F_2(Kt). \end{aligned}$$

If either $F_1$ or $F_2$ is non-decreasing then w.l.o.g. we can restrict to $K\in \mathbb {N}_{>0}$ and this relation is clearly reflexive and transitive. It has been introduced in [11, Chapter I, §3] and [17, Sect. 2.2, Def. 1]. In [11], $F_1$ and $F_2$ are called *comparable,* if either $F_1{\preceq _{\mathfrak {c}}}F_2$ or $F_2{\preceq _{\mathfrak {c}}}F_1$. In [17], when $F_1$, $F_2$ are related by (3.4), then $F_2$ has been called *stronger* than $F_1$.

For this relation we are gathering several equivalent reformulations.

#### Lemma 3.3

Let $F_1,F_2:[0,+\infty )\rightarrow [0,+\infty )$ be non-decreasing. Assume that either $F_1$ or $F_2$ is normalized, convex and tending to infinity as $t\rightarrow +\infty$. Then the following are equivalent:

(i) $$F_1{\preceq _{\mathfrak {c}}}F_2$$ holds.
(ii) We have that
  3.5 $$\begin{aligned} \exists \;K,K_1\ge 1\;\exists \;t_0>0\;\forall \;t\ge t_0:\;\;\;F_1(t)\le K_1F_2(Kt). \end{aligned}$$
(iii) We have that
  3.6 $$\begin{aligned} \exists \;C>0\;\exists \;K\ge 1\;\forall \;t\ge 0:\;\;\;F_1(t)\le F_2(Kt)+C. \end{aligned}$$
(iv) We have that
  3.7 $$\begin{aligned} \exists \;K,K_1\ge 1\;\exists \;C>0\;\forall \;t\ge 0:\;\;\;F_1(t)\le K_1F_2(Kt)+C. \end{aligned}$$

In particular, the above characterization applies if both $F_1$ and $F_2$ are N-functions.

#### Proof

$(i)\Rightarrow (ii)$ is trivial and $(ii)\Rightarrow (i)$ follows by (3.2): If $F_2$ is normalized and convex, then when given $K_1>1$ we put $t:=K_1^{-1}$ in (3.2) and hence $K_1F_2(u)\le F_2(K_1u)$ for all $u\ge 0$. Similarly, if $F_1$ is normalized and convex, then the assumption gives $K_1^{-1}F_1(t)\le F_2(Kt)$ and so $F_1(t)\le K_1^{-1}F_1(K_1t)\le F_2(KK_1t)$ for all $t\ge t_0$ holds which shows $F_1{\preceq _{\mathfrak {c}}}F_2$ when choosing $K_2:=KK_1$.

$(i)\Rightarrow (iii)$ is clear, since $F_2(t)\ge 0$ and $F_1$ is non-decreasing take e.g. $C:=F_1(t_0)$.

$(iii)\Rightarrow (ii)$ When given $C\ge 1$ then we have $F_2(Kt)+C\le 2F_2(Kt)$ for all sufficiently large *t* if $\lim _{t\rightarrow +\infty }F_2(t)=+\infty$. Thus (3.5) is verified with $K_1:=2$ and the same *K*. If $\lim _{t\rightarrow +\infty }F_1(t)=+\infty$, then by (3.6) also $\lim _{t\rightarrow +\infty }F_2(t)=+\infty$ and the rest follows as before.

$(iii)\Rightarrow (iv)$ is trivial and $(iv)\Rightarrow (iii)$ holds as $(ii)\Rightarrow (i)$. $\square$

This motivates the following definition, see [11, Chapter I, §3].

#### Definition 3.4

We call two functions $F_1$ and $F_2$ *equivalent,* written $F_1{\sim _{\mathfrak {c}}}F_2$, if $F_1{\preceq _{\mathfrak {c}}}F_2$ and $F_2{\preceq _{\mathfrak {c}}}F_1$.

In particular, for any N-function *F* we have that all $F_k:t\mapsto F(kt)$, $k>0$, are equivalent.

In [11, Thm. 13.2] it has been shown that $F_1{\sim _{\mathfrak {c}}}F_2$ if and only if $\mathcal {L}^{*}_{F_1}=\mathcal {L}^{*}_{F_2}$.

#### Remark 3.5

We comment on relation $\sim _{\mathfrak {c}}$ for given N-functions $F_1, F_2$ and their corresponding right-derivatives $f_1,f_2$ appearing in (3.1):

(i) On [11, p. 15] it is mentioned that if
  3.8 $$\begin{aligned} \exists \;b\in (0,+\infty ):\;\;\;\lim _{t\rightarrow +\infty }\frac{F_1(t)}{F_2(t)}=b, \end{aligned}$$
  then $F_1{\sim _{\mathfrak {c}}}F_2$ is valid. Indeed, this implication holds for any non-decreasing functions $F_1,F_2:[0,+\infty )\rightarrow [0,+\infty )$ such that either $F_1$ or $F_2$ is assumed to be convex and normalized: For any $0<a\le 1$ we clearly have $aF_2(u)\le F_2(u)$ and if $a>1$, then as in the proof of Lemma 3.3 the estimate (3.2) applied to $t:=a^{-1}$ gives $aF_2(u)\le F_2(au)$ for all $u\ge 0$. The proof for $F_1$ is analogous. In particular, (3.8) holds (with $b=1$) if $F_1(t)=F_2(t)$ for all *t* large.
(ii) Moreover, if $\lim _{t\rightarrow +\infty }F_i(t)=+\infty$, $i=1,2$, then (3.8) holds with $b=1$ if
  3.9 $$\begin{aligned} \exists \;C,D\ge 1\;\forall \;t\ge 0:\;\;\;F_1(t)-C\le F_2(t)\le F_1(t)+D. \end{aligned}$$
(iii) In [11, Lemma 3.1] it has been shown that $f_1{\preceq _{\mathfrak {c}}}f_2$ implies $F_1{\preceq _{\mathfrak {c}}}F_2$ and in [11, Lemma 3.2] that
  3.10 $$\begin{aligned} \exists \;b\in (0,+\infty ):\;\;\;\lim _{t\rightarrow +\infty }\frac{f_1(t)}{f_2(t)}=b \end{aligned}$$
  implies $$F_1{\sim _{\mathfrak {c}}}F_2$$.
(iv) Finally, let us characterize this relation for N-functions $F_1$, $F_2$ in terms of the right-derivatives $f_1$, $f_2$; see also [17, Sect. 2.2, Thm. 2] and [11, Lemma 3.1, p. 17–18]: We have $F_1{\preceq _{\mathfrak {c}}}F_2$ if and only if
  3.11 $$\begin{aligned} \exists \;k>1\;\exists \;t_1>0\;\forall \;t\ge t_1:\;\;\;f_1(t)\le kf_2(kt). \end{aligned}$$
  Note: $f_1{\preceq _{\mathfrak {c}}}f_2$ implies the above relation and since $f_2$ is non-decreasing, equivalently we can use in (3.11) the control $k_1f_2(k_2t)$ for some $k_1,k_2>1$. On the one hand, for all $x\ge t_1$ with $t_1$ denoting the value in (3.11)
  $$\begin{aligned} F_1(x)&=\int _0^xf_1(s)ds=\int _0^{t_1}f_1(s)ds+\int _{t_1}^xf_1(s)ds\le F_1(t_1)\\&\quad +\int _{t_1}^xkf_2(ks)ds\le F_1(t_1)+k\int _0^xf_2(ks)ds\\&=F_1(t_1)+k\int _0^{kx}f_2(u)\frac{du}{k}=F_1(t_1)+F_2(kx), \end{aligned}$$
  hence (3.6) is verified with $C:=2F_1(t_1)$ and $K:=k$. Conversely, let $x>0$ with $x\ge t_0/2$, $t_0$ from (3.4), and estimate as follows:
  $$\begin{aligned} xf_1(x)&\le \int _x^{2x}f_1(s)ds\le \int _0^{2x}f_1(s)ds=F_1(2x)\le F_2(2Kx)\\&=\int _0^{2Kx}f_2(s)ds\le 2Kxf_2(2Kx). \end{aligned}$$
  So (3.11) is verified with $k:=2K$ and $t_1:=t_0/2$.

#### Remark 3.6

In the theory of Orlicz classes also the following slightly different relation between functions is considered:

- Let $F_1,F_2$ be N-functions. In [11, Thm. 8.1], see also [1, Thm. 3.3], it has been shown that $\mathcal {L}_{F_1}(\Omega )\subseteq \mathcal {L}_{F_2}(\Omega )$ (as sets) if and only if
  3.12 $$\begin{aligned} \exists \;t_0>0\;\exists \;K\ge 1\;\forall \;t\ge t_0:\;\;\;F_2(t)\le KF_1(t), \end{aligned}$$
  i.e. $F_2(t)=O(F_1(t))$ as $t\rightarrow +\infty$. The sufficiency of (3.12) for having the inclusion $\mathcal {L}_{F_1}(\Omega )\subseteq \mathcal {L}_{F_2}(\Omega )$ is clear.
- Given $F_1,F_2:[0,+\infty )\rightarrow [0,+\infty )$ we write $F_1\preceq F_2$ if (3.12) holds and $F_1\sim F_2$ if $F_1\preceq F_2$ and $F_2\preceq F_1$. Note that this relation $\sim$ is precisely [11, (8.6)] and it is also the crucial one for the characterization of inclusions (resp. equalities) of classes in the ultradifferentiable weight function setting, see [15, Sect. 5]. Moreover, let us write $F_1\vartriangleleft F_2$ if $F_2(t)=o(F_1(t))$ as $t\rightarrow +\infty$.
- In view of (3.2), for given N-functions $F_1$, $F_2$ we have that $F_1\preceq F_2$ implies $F_2{\preceq _{\mathfrak {c}}}F_1$. For this implication one only requires that either $F_1$ or $F_2$ is normalized and convex, see the proof of $(ii)\Rightarrow (i)$ in Lemma 3.3. Consequently, if N-functions $F_1$ and $F_2$ are related by $F_1\sim F_2$, then they are also equivalent.

For the sake of completeness let us summarize more relations between (N-)functions mentioned in [17, Sect. 2.2, Def. 1]:

- $F_2$ is *essentially stronger* than $F_1$, if
  $$\begin{aligned} \forall \;K>0\;\exists \;t_0>0\;\forall \;t\ge t_0:\;\;\;F_1(t)\le F_2(Kt), \end{aligned}$$
  and write $F_1\preceq \preceq _{\mathfrak {c}}F_2$ for this relation which is, of course, stronger than $F_1{\preceq _{\mathfrak {c}}}F_2$. When taking $K:=1$ it also implies $F_2\preceq F_1$. Lemma 3.3 transfers to this relation and using this analogously to (*iv*) in Remark 3.5 one can prove that $F_1\preceq \preceq _{\mathfrak {c}}F_2$ if and only if
  3.13 $$\begin{aligned} \forall \;k>1\;\exists \;t_1>0\;\forall \;t\ge t_1:\;\;\;f_1(t)\le kf_2(kt). \end{aligned}$$
- $F_2$ is *completely stronger* than $F_1$, if
  $$\begin{aligned} \forall \;\epsilon>0\;\;\exists \;K>0\;\exists \;t_0>0\;\forall \;t\ge t_0:\;\;\;F_1(t)\le K F_2(\epsilon t). \end{aligned}$$
  The choice $\epsilon :=1$ implies again $F_2\preceq F_1$.
- $F_2$ is *increasing more rapidly* than $F_1$, if
  $$\begin{aligned} \forall \;\epsilon>0\;\exists \;\delta>0\;\exists \;t_0>0\;\forall \;t\ge t_0:\;\;\;\frac{1}{\delta }F_1(\delta t)\le \epsilon F_2(t). \end{aligned}$$
  If $F_2\vartriangleleft F_1$ is valid, then $F_2$ is increasing more rapidly than $F_1$ by the uniform choice $\delta :=1$ for all $\epsilon >0$.

### From weight sequences to associated N-functions

Let $M\in {\mathcal{L}\mathcal{C}}$ be given. When rewriting (2.3) we obtain for all $t\ge 0$ (note that $\mu _1\ge 1$):

3.14 $$\begin{aligned} \varphi _{\omega _M}(t)= & {} \omega _M(e^t)=\int _{\mu _1}^{e^t}\frac{\Sigma _M(s)}{s}ds =\int _{\log (\mu _1)}^t\frac{\Sigma _M(e^u)}{s}sdu\nonumber \\= & {} \int _{\log (\mu _1)}^t\Sigma _M(e^u)du=\int _0^t\Sigma _M(e^u)du, \end{aligned}$$

since $\Sigma _M(e^u)=0$ for $0\le u<\log (\mu _1)$. This formula should be compared with [11, Thm. 1.1, (1.10)]. $\Sigma _M\circ \exp$ is right-continuous, non-decreasing and clearly $\lim _{t\rightarrow +\infty }\Sigma _M(e^t)=+\infty$.

Recall that $\omega _M\in {\mathcal {W}_0}$ and so $\varphi _{\omega _M}$ is convex, $t\mapsto \frac{\varphi _{\omega _M}(t)}{t}$ is non-decreasing with $\lim _{t\rightarrow +\infty }\frac{\varphi _{\omega _M}(t)}{t}=+\infty$ and finally $\varphi _{\omega _M}(0)=0$ is valid, see e.g. [2, Rem. 1.3, Lemma 1.5] and also [11, p. 7].

#### Remark 3.7

However, requirement (*II*) cannot be achieved for $\Sigma _M\circ \exp$ for any $M\in {\mathcal{L}\mathcal{C}}$: If $M_1=M_0(=1)$, and so $\mu _1=1$, then (2.2) yields $\Sigma _M(e^0)=\Sigma _M(\mu _1)\ge 1\ne 0$. If $M_1>M_0\Leftrightarrow \mu _1>1$, then $\Sigma _M(e^t)=0$ for all $0\le t<\log (\mu _1)$.

Finally remark that, if *M* is log-convex with $\lim _{j\rightarrow +\infty }(M_j)^{1/j}=+\infty$ but such that normalization fails, then $0<\mu _1<1$ and so $\Sigma _M(e^t)\ge 1$ for any $t\ge 0$. Thus also in this case the first requirement in (*II*) is violated.

This failure is related to the fact that the first property in (3.3) and $\frac{\varphi _{\omega _M}(t)}{t}>0$ for all $t>0$ are not satisfied automatically for $\varphi _{\omega _M}$, see the proofs and arguments in [11, Chapter I, §1, 5, p. 8–9]. Thus $\varphi _{\omega _M}$ is formally not an N-function according to Definition 3.1.

In order to overcome this technical problem we recall the following notion, see [11, Chapter I, §3, 3, p. 16]:

#### Definition 3.8

A convex function *Q* is called the principal part of an N-function *F* if

$$\begin{aligned} \exists \;t_0>0\;\forall \;t\ge t_0:\;\;\;Q(t)=F(t). \end{aligned}$$

We have the following result, see [11, Thm. 3.3] and the proof there:

#### Theorem 3.9

Let $Q:[0,+\infty )\rightarrow [0,+\infty )$ be a convex function such that $\lim _{t\rightarrow +\infty }\frac{Q(t)}{t}=+\infty$. Then there exists an N-function *F* such that *Q* is the principal part of *F*.

More precisely, we even get that

3.15 $$\begin{aligned} \exists \;t_0>0\;\forall \;t\ge t_0:\;\;\;f(t)=q(t), \end{aligned}$$

with *f* denoting the function appearing in (3.1) of *F* and *q* denoting the non-decreasing and right-continuous function appearing in the representation

3.16 $$\begin{aligned} Q(t)=\int _a^tq(s)ds, \end{aligned}$$

see [11, (1.10)]. Here $a\ge 0$ is such that $Q(a)=0$ and we have $t_0>a$.

#### Proposition 3.10

Let *Q* be a convex function such that $\lim _{t\rightarrow +\infty }\frac{Q(t)}{t}=+\infty$ and let *F* be the N-function according to Theorem 3.9. Then we get

3.17 $$\begin{aligned} \exists \;C,D\ge 1\;\forall \;t\ge 0:\;\;\;Q(t)-C\le F(t)\le Q(t)+D, \end{aligned}$$

cf. (3.9). This relation implies $\lim _{t\rightarrow +\infty }\frac{F(t)}{Q(t)}=1$ and so both $F{\sim _{\mathfrak {c}}}Q$ and $F\sim Q$ holds.

#### Proof

More generally, when for given functions $F_1, F_2:[0,+\infty )\rightarrow [0,+\infty )$ we get $F_1(t)=F_2(t)$ for all *t* large then we have $F_1(t)\le F_2(t)+C$, $F_2(t)\le F_1(t)+D$ for all $t\ge 0$ with $C:=\max \{F_1(t): 0\le t\le t_0\}$ and $D:=\max \{F_2(t): 0\le t\le t_0\}$.

Hence (3.17) follows by Theorem 3.9 (recall Definition 3.8).

Note that *Q* is convex but normalization for *Q* (i.e. $a=0$ in (3.16)) is not guaranteed in general. $\square$

#### Remark 3.11

Conversely, each convex function $Q:[0,+\infty )\rightarrow [0,+\infty )$ admitting the representation (3.16) for a non-decreasing and right-continuous function *q* with $q(t)\rightarrow +\infty$ satisfies also $\lim _{t\rightarrow +\infty }\frac{Q(t)}{t}=+\infty$. This holds since for all $t\ge a$ (see the proof of (1.16) in [11, Chapter I, §1, p. 7]):

$$\begin{aligned} Q(2t)=\int _a^{2t}q(s)ds\ge \int _t^{2t}q(s)ds\ge q(t)t. \end{aligned}$$

In particular, when applying these results to $Q=\varphi _{\omega _M}$ we get the following consequence:

#### Corollary 3.12

Let $M\in {\mathcal{L}\mathcal{C}}$ be given. Then there exists an N-function $F_M$ such that $\varphi _{\omega _M}$ is the principal part of $F_M$ and so

3.18 $$\begin{aligned} \exists \;C,D\ge 1\;\forall \;t\ge 0:\;\;\;\varphi _{\omega _M}(t)-C\le F_M(t)\le \varphi _{\omega _M}(t)+D. \end{aligned}$$

This implies $\varphi _{\omega _M}\sim F_M$ and hence also $\varphi _{\omega _M}{\sim _{\mathfrak {c}}}F_M$.

Moreover, if $f_M$ denotes the function appearing in the representation (3.1) of $F_M$, then we even get

3.19 $$\begin{aligned} \exists \;t_0>0\;\forall \;t\ge t_0:\;\;\;f_M(t)=\Sigma _M(e^t). \end{aligned}$$

In view of this equality we call $\Sigma _M\circ \exp$ the principal part of $f_M$ (see [11, p. 18]).

#### Proof

We can apply Theorem 3.9 to $Q\equiv \varphi _{\omega _M}$ because $\lim _{t\rightarrow +\infty }\frac{\varphi _{\omega _M}(t)}{t}=\lim _{s\rightarrow +\infty }\frac{\omega _M(s)}{\log (s)}=+\infty$ by $(\omega _3)$ (recall [9, Lemma 3.1] and the citations there). In fact $(\omega _3)$ for $\omega _M$ is precisely [11, (3.6)] for $Q=\varphi _{\omega _M}$.

(3.18) follows by Proposition 3.10, and (3.19) holds by taking into account the representation (3.14).

Note that by normalization of *M* we get $\varphi _{\omega _M}(0)=\omega _M(1)=0$ and so in (3.16) we have $a=0$ and $q=\Sigma _M\circ \exp$. $\square$

The following is an immediate consequence of (3.18):

#### Corollary 3.13

Let $M,L\in {\mathcal{L}\mathcal{C}}$ be given. Then $F_M{\preceq _{\mathfrak {c}}}F_L$ if and only if $\varphi _{\omega _M}{\preceq _{\mathfrak {c}}}\varphi _{\omega _L}$ and $F_M\preceq F_L$ if and only if $\varphi _{\omega _M}\preceq \varphi _{\omega _L}$.

Moreover, also all further relations from [17, Sect. 2.2, Def. 1] hold between $F_M$, $F_L$ if and only if they are valid between $\varphi _{\omega _M}$, $\varphi _{\omega _L}$.

#### Definition 3.14

Let $M\in {\mathcal{L}\mathcal{C}}$ be given. Then the N-function $F_M$ from Corollary 3.12 is called the associated N-function.

We close this section by commenting on the relation between $\varphi _{\omega _M}$ and other notions of defining functions in the Orlicz setting.

#### Remark 3.15

As seen above, for any given $M\in {\mathcal{L}\mathcal{C}}$ we cannot expect that $\varphi _{\omega _M}$ is formally an N-function. On the other hand $\varphi _{\omega _M}$ can be used to illustrate the differences between appearing definitions for Orlicz classes in the literature. In [14] an exhaustive study is provided and the different notions and conditions for the defining functions are compared, see also the literature citations there.

- $\varphi _{\omega _M}$ coincides with an N-function (with $F_M$) for sufficiently large values.
- For any $M\in {\mathcal{L}\mathcal{C}}$ the function $\varphi _{\omega _M}$ is always a *Young function*, see [14, Def. 1.4] and [17, Sect. 1.3, p. 6]: $\varphi _{\omega _M}$ is convex, satisfies $\varphi _{\omega _M}(0)=\omega _M(1)=0$ by normalization and $\varphi _{\omega _M}(t)\rightarrow +\infty$ as $t\rightarrow +\infty$ (and it can be extend to $\mathbb {R}$ in an even way). Note that formally for Young functions *F* it is allowed that $F(a)=+\infty$ for some $a\in \mathbb {R}$.
- $\varphi _{\omega _M}$ is a *strong Young function* (see [14, Def. 1.7]) if and only if $\mu _1=1$: Continuity is clear and $\varphi _{\omega _M}(t)>0$ for all $t>0$ follows if and only if $\Sigma _M(e^t)>0$ for all $t\ge 0$, see (3.14). This is clearly equivalent to $\mu _1=1$.
- Finally, $\varphi _{\omega _M}$ is always an *Orlicz function* (see [14, Def. 1.9]), since $\varphi _{\omega _M}$ is never identically zero or infinity (follows again by (3.14)).
- Consequently, $\varphi _{\omega _M}$ provides (counter-)examples for the first two strict implications in [14, Cor. 2.7], see [14, Cor. 2.8]: Each N-function is a strong Young function and each strong Young function is an Orlicz function but each implication cannot be reversed in general; one can take $M\in {\mathcal{L}\mathcal{C}}$ with $\mu _1=1$ for the first and $M\in {\mathcal{L}\mathcal{C}}$ with $\mu _1>1$ for the second part.

## Comparison between associated N-functions

The goal of this section is to give a connection resp. comparison between the growth relation $\preceq$ for weight sequences, crucially appearing in the theory of ultradifferentiable and ultraholomorphic functions, and the previously defined relations $\preceq _{\mathfrak {c}}$ and $\preceq$ for (associated) N-functions.

### Main statements

The first main result establishes a characterization of relation $F_M{\preceq _{\mathfrak {c}}}F_L$ in terms of a growth comparison between *M* and *L*. However, the characterization is not given via $\preceq$ but expressed in terms of the corresponding sequences of quotients $\mu =(\mu _j)_{j\in \mathbb {N}}$ and $\lambda =(\lambda _j)_{j\in \mathbb {N}}$. In explicit applications and for constructing weight sequences *M* it is often convenient to start with $\mu$. Note that when involving $\mu$ we get automatically growth conditions for the counting function $\Sigma _M$ as well.

#### Theorem 4.1

Let $M,L\in {\mathcal{L}\mathcal{C}}$ be given. Then the following are equivalent:

- We have that
  4.1 $$\begin{aligned} \exists \;A\ge 1\;\exists \;k>0\;\exists \;t_0>0\;\forall \;t\ge t_0:\;\;\;\Sigma _M(t)\le A\Sigma _L(t^k). \end{aligned}$$
- We have that
  4.2 $$\begin{aligned} \exists \;B\ge 1\;\exists \;k>0\;\exists \;j_0\in \mathbb {N}_{>0}\;\forall \;j\ge j_0:\;\;\;\lambda _{\lceil j/B\rceil }\le \mu _j^k. \end{aligned}$$
- We have that $F_M{\preceq _{\mathfrak {c}}}F_L$ (equivalently $\varphi _{\omega _M}{\preceq _{\mathfrak {c}}}\varphi _{\omega _L}$) holds.
- We have that (3.11) holds between $f_M$ and $f_L$, i.e.
  $$\begin{aligned} \exists \;k>1\;\exists \;t_1>0\;\forall \;t\ge t_1:\;\;\;f_M(t)\le kf_L(kt). \end{aligned}$$

The proof shows that we can take $A=B=k$ in (*a*) and (*b*) and the result becomes trivial for $M=L$ (set $A=B=k=1$).

The analogous characterization for $F_M\preceq \preceq _{\mathfrak {c}}F_L$ is obtained when replacing (4.1) by (3.13) and so (4.2) by the condition

$$\begin{aligned} \forall \;k>0\;\exists \;j_0\in \mathbb {N}_{>0}\;\forall \;j\ge j_0:\;\;\;\lambda _{\lceil j/k\rceil }\le \mu _j^k. \end{aligned}$$

#### Proof

$(a)\Rightarrow (b)$ Let $j_0\in \mathbb {N}_{>0}$ be minimal to ensure $\mu _{j_0}\ge t_0$ (note that $\lim _{j\rightarrow +\infty }\mu _j=+\infty$). For any $t\ge \mu _{j_0}$ we have $\mu _j\le t<\mu _{j+1}$ for some $j\in \mathbb {N}_{>0}$, $j\ge j_0$. Then by assumption $j=\Sigma _M(t)\le A\Sigma _L(t^k)\Leftrightarrow \Sigma _L(t^k)\ge \frac{j}{A}$ and so $t^k\ge \lambda _{\lceil j/A\rceil }$ (note that $\Sigma _L(t^k)\in \mathbb {N}$). When taking $t:=\mu _j$ we get (4.2) with $B:=A$ and the same $k>0$ for all $j\ge j_0$ with $\mu _j<\mu _{j+1}$.

If $j\ge j_0$ with $\mu _j=\mu _{j+1}$, then $\mu _j=\mu _{j+1}=\dots =\mu _{j+d}<\mu _{j+d+1}$ for some $d\in \mathbb {N}_{>0}$ and thus $j+d=\Sigma _M(t)$ for $\mu _{j+d}\le t<\mu _{j+d+1}$. This yields $\Sigma _L(t^k)\ge \frac{j+d}{A}$, i.e. $t^k\ge \lambda _{\lceil (j+d)/A\rceil }\ge \lambda _{\lceil (j+i)/A\rceil }$ for all $0\le i\le d$. Put $t:=\mu _{j+d}(=\dots =\mu _j)$ in order to verify (4.2) with $B:=A$ and the same $k>0$ for all $j\ge j_0$.

$(b)\Rightarrow (a)$ Let $t\ge 0$ be such that $\mu _j\le t<\mu _{j+1}$ for some $j\ge j_0$. Then $\mu _j^k\le t^k<\mu ^k_{j+1}$ and so $t^k\ge \mu _j^k\ge \lambda _{\lceil j/B\rceil }$ which gives $\Sigma _M(t)=j$ and $\lceil j/B\rceil \le \Sigma _L(t^k)$. Thus (4.1) follows when $j\le A j/B\le A\lceil j/B\rceil$ is ensured. So we can take $A:=B$, the same $k>0$ and $t_0:=\mu _{j_0}$.

$(a)\Leftrightarrow (c)$ First, (4.1) precisely means that $\Sigma _M(e^s)\le A\Sigma _L(e^{sk})$ for some $A\ge 1$, $k>0$ and all sufficiently large *s*. Thus the desired equivalence holds by taking into account the representation (3.14), following the estimates in (*iv*) in Remark 3.5 with $f_1$ being replaced by $\Sigma _M\circ \exp$ and $f_2$ by $\Sigma _L\circ \exp$ ((4.1) is precisely (3.11) for these choices), and finally using Corollary 3.12.

$(a)\Leftrightarrow (d)$ This holds by (3.19). $\square$

We continue with the following result providing a complete characterization for the relation $\preceq$ between associated N-functions.

#### Theorem 4.2

Let $M,L\in {\mathcal{L}\mathcal{C}}$ be given. Then the following are equivalent:

(i) The associated N-functions satisfy $F_M\preceq F_L$.
(ii) The functions $\varphi _{\omega _M}$ and $\varphi _{\omega _L}$ satisfy $\varphi _{\omega _M}\preceq \varphi _{\omega _L}$.
(iii) The sequences *M* and *L* are related by
  4.3 $$\begin{aligned} \exists \;A\ge 1\;\exists \;c\in \mathbb {N}_{>0}\;\forall \;j\in \mathbb {N}:\;\;\;M_j\le A(L_{cj})^{1/c}. \end{aligned}$$

#### Proof

$(i)\Leftrightarrow (ii)$ holds by Corollary 3.13.

$(ii)\Leftrightarrow (iii)$ First note that $\varphi _{\omega _M}\preceq \varphi _{\omega _L}$ precisely means

$$\begin{aligned} \exists \;K,D\ge 1\;\forall \;t\ge 0:\;\;\;\omega _L(e^t)=\varphi _{\omega _L}(t)\le K\varphi _{\omega _M}(t)+D=K\omega _M(e^t)+D. \end{aligned}$$

Since $\omega _M(t)=\omega _L(t)=0$ for all $0\le t\le 1$ by normalization of *M* and *L* we get $\omega _L(t)\le K\omega _M(t)+D$ for all $t\ge 0$, i.e. $\omega _L(t)=O(\omega _M(t))$ and so $\omega _M\preceq \omega _L$. Similarly, $\omega _M\preceq \omega _L$ implies $\varphi _{\omega _M}\preceq \varphi _{\omega _L}$ as well.

Consequently, the desired equivalence $(ii)\Leftrightarrow (iii)$ follows by the first part in [4, Lemma 6.5]. $\square$

Next let us gather some more immediate consequences concerning relation $\preceq _{\mathfrak {c}}$.

#### Remark 4.3

Let $M,L\in {\mathcal{L}\mathcal{C}}$ be given.

(i) If $L\le M$, then by definition $\omega _M(t)\le \omega _L(t)$ for all $t\ge 0$ and so $\varphi _{\omega _M}(t)\le \varphi _{\omega _L}(t)$ for all $t\ge 0$. In view of Corollary 3.12 we get $F_M{\preceq _{\mathfrak {c}}}F_L$.
(ii) More generally, if $L{\preceq }M$, then by definition and since $M_0=N_0=1$ we have $\omega _M(t)\le \omega _L(ht)$ for some $h>1$ and all $t\ge 0$. Hence
  $$\begin{aligned}{} & {} \exists \;s_h>0\;\forall \;s\ge s_h:\;\;\;\varphi _{\omega _M}(s)=\omega _M(e^s)\le \omega _L(e^{s+\log (h)})\\{} & {} \quad =\varphi _{\omega _L}(s+\log (h))\le \varphi _{\omega _L}(sh), \end{aligned}$$
  since $\varphi _{\omega _L}$ is non-decreasing and $s+\log (h)\le sh\Leftrightarrow \log (h)\le s(h-1)$ for all *s* large enough. This verifies $\varphi _{\omega _M}{\preceq _{\mathfrak {c}}}\varphi _{\omega _L}$ and Corollary 3.12 implies again $F_M{\preceq _{\mathfrak {c}}}F_L$.
(iii) Consequently, equivalent weight sequences yield equivalent associated N-functions and, in particular, this applies to the situation described in Remark 2.2.
(iv) However, the converse implication in (*iii*) is not true in general. For this recall that by (2.4) we get
  $$\begin{aligned} \forall \;\ell >0\;\forall \;t\ge 0:\;\;\;\varphi _{\omega _{M^{\ell }}}(t)=\omega _{M^{\ell }}(e^t)=\ell \omega _M(e^{t/\ell })=\ell \varphi _{\omega _M}(t/\ell ). \end{aligned}$$
  Then, by following the arguments in (*i*) in Remark 3.5, we see that
  4.4 $$\begin{aligned} \forall \;\ell >0:\;\;\;\varphi _{\omega _M}{\sim _{\mathfrak {c}}}\varphi _{\omega _{M^{\ell }}}, \end{aligned}$$
  hence by Corollary 3.12 also $F_M{\sim _{\mathfrak {c}}}F_{M^{\ell }}$ for any $\ell >0$. However, for $\ell \ne 1$ the sequences *M* and $M^{\ell }$ are not equivalent since $\lim _{j\rightarrow +\infty }(M_j)^{1/j}=+\infty$.
(v) In particular, (*iv*) applies to the Gevrey-sequence $M\equiv G^s$, $s>0$. It is known that $\omega _{G^s}\sim t\mapsto t^{1/s}$, i.e. $\varphi _{\omega _{G^s}}\sim t\mapsto e^{t/s}$ (see the proof of Theorem 4.2). So (4.4) is verified but clearly $G^s$ is not equivalent to $G^{s'}$ if $s\ne s'$.

The next result provides a second characterization for $F_M{\preceq _{\mathfrak {c}}}F_L$ in terms of a growth relation between *M* and *L* directly.

#### Theorem 4.4

Let $M,L\in {\mathcal{L}\mathcal{C}}$ be given. Consider the following assertions:

(i) $$L{\preceq }M$$ is valid.
(ii) The associated N-functions $F_M$ and $F_L$ (see Corollary 3.12) satisfy
  $$\begin{aligned} F_M{\preceq _{\mathfrak {c}}}F_L, \end{aligned}$$
  equivalently $\varphi _{\omega _M}{\preceq _{\mathfrak {c}}}\varphi _{\omega _L}$ is valid.
(iii) The sequences *M* and *L* satisfy
  4.5 $$\begin{aligned} \exists \;c\in \mathbb {N}_{>0}\;\exists \;A\ge 1\;\forall \;j\in \mathbb {N}:\;\;\;L_j\le AM_{cj}. \end{aligned}$$

Then $(i)\Rightarrow (ii)\Rightarrow (iii)$ holds. If either *M* or *L* has in addition $({\text {mg}})$, then also $(iii)\Rightarrow (ii)$.

By (*iv*) in Remark 4.3 the implication $(i)\Rightarrow (ii)$ cannot be reversed in general.

#### Proof

$(i)\Rightarrow (ii)$ This is shown in (*i*), (*ii*) in Remark 4.3.

$(ii)\Rightarrow (iii)$ By Lemma 3.3 relation $F_M{\preceq _{\mathfrak {c}}}F_L$ is equivalent to

$$\begin{aligned} \exists \;K\ge 1\;\exists \;C\ge 1\;\forall \;t\ge 0:\;\;\;F_M(t)\le F_L(Kt)+C, \end{aligned}$$

and so by Corollary 3.12 (take w.l.o.g. $K\in \mathbb {N}_{>0}$)

$$\begin{aligned} \exists \;K\in \mathbb {N}_{>0}\;\exists \;D\ge 1\;\forall \;t\ge 0:\;\;\;\omega _M(e^t)=\varphi _{\omega _M}(t)\le \varphi _{\omega _L}(Kt)+D=\omega _L(e^{tK})+D. \end{aligned}$$

We set $s:=e^t$ and hence this is equivalent to

$$\begin{aligned} \exists \;K\in \mathbb {N}_{>0}\;\exists \;D\ge 1\;\forall \;s\ge 1:\;\;\;\omega _M(s)\le \omega _L(s^{K})+D. \end{aligned}$$

Recall that $M,L\in {\mathcal{L}\mathcal{C}}$ implies (by normalization) $\omega _M(s)=\omega _L(s)=0$ for all $s\in [0,1]$ and so the previous estimate holds for any $s\ge 0$ (with the same constants). Thus (2.5) yields for all $j\in \mathbb {N}$:

$$\begin{aligned} M_{Kj}&=\sup _{t\ge 0}\frac{t^{Kj}}{\exp (\omega _M(t))}\ge \frac{1}{e^{D}}\sup _{t\ge 0}\frac{t^{Kj}}{\exp (\omega _L(t^{K}))}=\frac{1}{e^{D}}\sup _{s\ge 0}\frac{s^j}{\exp (\omega _L(s))}=\frac{1}{e^{D}}L_j, \end{aligned}$$

and we are done when taking $c:=K$ and $A:=e^{D}$.

$(iii)\Rightarrow (ii)$ Assume that $({\text {mg}})$ holds for *M*. By assumption (4.5) and an iterated application of $({\text {mg}})$ we get

4.6 $$\begin{aligned} \exists \;c\in \mathbb {N}_{>0}\;\exists \;A,B\ge 1\;\forall \;k\in \mathbb {N}:\;\;\;L_k\le AM_{ck}\le AB^kM_k^c, \end{aligned}$$

thus by definition of associated weight functions $\omega _{M^c}(t)\le \omega _L(Bt)+\log (A)$ for all $t\ge 0$. Consequently, since $\varphi _{\omega _L}(t)\rightarrow +\infty$ and by (3.2) we get for all *t* large enough

$$\begin{aligned} \varphi _{\omega _{M^c}}(t)\le \varphi _{\omega _L}(t+\log (B))+\log (A)\le \varphi _{\omega _L}(2t)+\log (A)\le 2\varphi _{\omega _L}(2t)\le \varphi _{\omega _L}(4t), \end{aligned}$$

hence $\varphi _{\omega _{M^c}}{\preceq _{\mathfrak {c}}}\varphi _{\omega _L}$. By (4.4) we have that $\varphi _{\omega _{M^c}}{\sim _{\mathfrak {c}}}\varphi _{\omega _M}$ and so $\varphi _{\omega _M}{\preceq _{\mathfrak {c}}}\varphi _{\omega _L}$ is verified. Corollary 3.12 yields the conclusion.

If *L* has in addition $({\text {mg}})$, then by (4.5) and iterating $({\text {mg}})$ first we get

$$\begin{aligned} \exists \;c\in \mathbb {N}_{>0}\;\exists \;A,B_1\ge 1\;\forall \;j\in \mathbb {N}:\;\;\;L_{cj}\le B_1^{cj}L_j^c\le A^cB_1^{cj}M^c_{cj}. \end{aligned}$$

Thus (4.6) is verified for all $k\in \mathbb {N}$ with $k=cj$, $j\in \mathbb {N}$ arbitrary. For the remaining cases let *k* with $cj<k<cj+c$ for some $j\in \mathbb {N}$ and then, since both *M* and *L* are also non-decreasing, we get for some $C\ge 1$

$$\begin{aligned} L_k&\le L_{cj+c}\le C^{c(j+1)}L_cL_{cj}\le C^{c(j+1)}L_c A^cB_1^{cj}M^c_{cj}\le (AC)^cL_c(B_1C)^kM^c_k. \end{aligned}$$

Summarizing, (4.6) is verified for all $k\in \mathbb {N}$ and the rest follows as above. $\square$

Thus we have the following characterization:

#### Corollary 4.5

Let $M,L\in {\mathcal{L}\mathcal{C}}$ be given. Assume that either *M* or *L* has in addition $({\text {mg}})$, then the following are equivalent:

(i) The associated N-functions $F_M$ and $F_L$ are equivalent.
(ii) The functions $\varphi _{\omega _M}$ and $\varphi _{\omega _L}$ are equivalent.
(iii) The sequences *M* and *L* satisfy
  $$\begin{aligned} \exists \;c,d\in \mathbb {N}_{>0}\;\exists \;A,B\ge 1\;\forall \;j\in \mathbb {N}:\;\;\;M_j\le AL_{cj},\;\;\;\;\;L_j\le BM_{dj}. \end{aligned}$$

The proof of $(ii)\Rightarrow (iii)$ in Theorem 4.4 transfers to relation $\preceq \preceq _{\mathfrak {c}}$ immediately: If $F_M\preceq \preceq _{\mathfrak {c}}F_L,$ equivalently if $\varphi _{\omega _M}\preceq \preceq _{\mathfrak {c}}\varphi _{\omega _L}$, then the sequences *M* and *L* satisfy

$$\begin{aligned} \forall \;c\in \mathbb {N}_{>0}\;\exists \;A\ge 1\;\forall \;j\in \mathbb {N}:\;\;\;L_j\le AM_{cj}. \end{aligned}$$

We finish by comparing the characterizing conditions for *M* and *L* in the previous results.

#### Remark 4.6

Let $M,L\in {\mathcal{L}\mathcal{C}}$ be given.

- We have $L_j\ge 1$ for all $j\in \mathbb {N}$ and so (4.3) implies (4.5) (with *M*, *L* interchanged).
- On the other hand, recall that by (3.2) we get that $F_M\preceq F_L$ implies $F_L{\preceq _{\mathfrak {c}}}F_M$. Summarizing,
  4.7 $$\begin{aligned} (4.3)\Longleftrightarrow F_M\preceq F_L\Longrightarrow F_L{\preceq _{\mathfrak {c}}}F_M\Longrightarrow (4.5), \end{aligned}$$
  and the last implication can be reversed provided that either *M* or *L* has in addition $({\text {mg}})$.

### On sufficiency conditions

We want to find some sufficient conditions for given sequences *M*, *L* in order to ensure $F_M{\sim _{\mathfrak {c}}}F_L$. More precisely, the aim is to ensure an asymptotic behavior of the counting functions near infinity (see (4.8)) which might be important for applications in the ultradifferentiable setting as well.

#### Lemma 4.7

Let $M,L\in {\mathcal{L}\mathcal{C}}$ be given. Assume that

4.8 $$\begin{aligned} \exists \;b\in (0,+\infty ):\;\;\;\lim _{t\rightarrow +\infty }\frac{\Sigma _M(t)}{\Sigma _L(t)}=b. \end{aligned}$$

Then we get $F_M{\sim _{\mathfrak {c}}}F_L$ (equivalently $\varphi _{\omega _M}{\sim _{\mathfrak {c}}}\varphi _{\omega _L}$).

*Note:* For $M=L$ property (4.8) holds trivially with $b=1$.

#### Proof

By (3.19) and (4.8) we have $\lim _{t\rightarrow +\infty }\frac{f_M(t)}{f_L(t)}=b>0$ and so [11, Lemma 3.2] yields $F_M{\sim _{\mathfrak {c}}}F_L$. $\square$

Let us now study condition (4.8) in more detail.

#### Lemma 4.8

Let $M,L\in {\mathcal{L}\mathcal{C}}$ be given. Assume that

4.9 $$\begin{aligned}{} & {} \exists \;c,j_0\in \mathbb {N}_{>0}\;\forall \;j\ge j_0,\;\text {s.th.}\;\mu _j<\mu _{j+1},\;\exists \;d_j\in \mathbb {N}_{>0}:\nonumber \\{} & {} \quad \lambda _{cj}\le \mu _j<\mu _{j+1}\le \lambda _{cj+d_j}, \end{aligned}$$

with $d_j\in \mathbb {N}_{>0}$ such that $\lim _{j\rightarrow +\infty }\frac{d_j}{j}=0$. Then (4.8) holds (with $b=\frac{1}{c}$).

#### Proof

For all $t\ge \mu _{j_0}$ we find $j\ge j_0$ such that $\mu _j\le t<\mu _{j+1}$. Then $\Sigma _M(t)=j$ and by (4.9) we get $cj\le \Sigma _L(t)<cj+d_j$. Thus

$$\begin{aligned} \frac{j}{cj+d_j}<\frac{\Sigma _M(t)}{\Sigma _L(t)}\le \frac{j}{cj},\;\;\;\forall \;\mu _j\le t<\mu _{j+1},\;j\ge j_0, \end{aligned}$$

and since $\lim _{j\rightarrow +\infty }\frac{d_j}{j}=0$ we get (4.8) with $b:=\frac{1}{c}$. $\square$

Note that it is enough to require the existence of a sequence $d_j$ for *j* such that $\mu _j<\mu _{j+1}$: If $j\ge j_0$ and $\mu _j=\mu _{j+1}=\dots =\mu _{j+\ell }<\mu _{j+\ell +1}$ for some $\ell \in \mathbb {N}_{>0}$, then by (4.9) we get

$$\begin{aligned} \lambda _{cj}\le \lambda _{cj+c\ell }\le \mu _j=\mu _{j+1}=\dots =\mu _{j+\ell }<\mu _{j+\ell +1}\le \lambda _{cj+c\ell +d_{j+\ell }}, \end{aligned}$$

and so for *t* with $\mu _{j+\ell }\le t<\mu _{j+\ell +1}$ we have $\Sigma _M(t)=j+\ell$ and $cj+c\ell =c(j+\ell )\le \Sigma _L(t)<cj+c\ell +d_{j+\ell }=c(j+\ell )+d_{j+\ell }$ yielding the same estimate as above. (On the other hand, by switching to an equivalent sequence, we can assume $\mu _j<\mu _{j+1}$ for all $j\in \mathbb {N}_{>0}$, see Remark 2.2.)

We prove now the following characterization for (4.8).

#### Lemma 4.9

Let $M,L\in {\mathcal{L}\mathcal{C}}$ be given such that $\mu _j<\mu _{j+1}$ for all $j\in \mathbb {N}_{>0}$. Then the following are equivalent:

(i) We have that
  4.10 $$\begin{aligned} \exists \;b\in (0,+\infty ):\;\;\;\lim _{t\rightarrow +\infty }\frac{\Sigma _M(t)}{\Sigma _L(t)}=b, \end{aligned}$$
  i.e. (4.8).
(ii) We have that
  4.11 $$\begin{aligned}{} & {} \exists \;b\in (0,+\infty )\;\exists \;d\in \mathbb {N}\;\forall \;0<c_1<b<c_2\;\exists \;j_0\in \mathbb {N}_{>0}\;\nonumber \\{} & {} \quad \forall j\ge j_0:\;\;\;\lambda _{\lceil j/c_2\rceil -d}\le \mu _j<\lambda _{\lceil j/c_1\rceil +d}. \end{aligned}$$
(iii) We have that
  4.12 $$\begin{aligned}{} & {} \exists \;b\in (0,+\infty )\;\forall \;0<c_1<b<c_2\;\exists \;j_0\in \mathbb {N}_{>0}\;\forall \;j\ge j_0\nonumber \\{} & {} \quad \exists \;d_j\in \mathbb {N}_{>0}:\;\;\;\lambda _{\lceil j/c_2\rceil -d_j}\le \mu _j<\lambda _{\lceil j/c_1\rceil +d_j}, \end{aligned}$$
  with $d_j\in \mathbb {N}_{>0}$ such that $\lim _{j\rightarrow +\infty }\frac{d_j}{j}=0$.

The proof shows that in all assertions the value *b* is the same.

#### Proof

$$(i)\Rightarrow (ii)$$ By assumption,

$$\begin{aligned} \forall \;\epsilon>0\;\exists \;t_{\epsilon }>0\;\forall \;t\ge t_{\epsilon }:\;\;\;\Sigma _L(t)(b-\epsilon )<\Sigma _M(t)<(b+\epsilon )\Sigma _L(t), \end{aligned}$$

thus we find $j_{\epsilon }\in \mathbb {N}_{>0}$ large such that $\mu _{j_{\epsilon }}\ge t_{\epsilon }$ so that for all *t* with $\mu _j\le t<\mu _{j+1}$ for some $j\ge j_{\epsilon }$ we get

4.13 $$\begin{aligned} \Sigma _L(t)(b-\epsilon )<j=\Sigma _M(t)<(b+\epsilon )\Sigma _L(t). \end{aligned}$$

The first estimate in (4.13) yields $\Sigma _L(t)<\frac{j}{b-\epsilon }\le \lceil \frac{j}{b-\epsilon }\rceil$ and since $\Sigma _L(t)\in \mathbb {N}$ we have $\Sigma _L(t)\le \lceil \frac{j}{b-\epsilon }\rceil -1$. Let now $c_1<b$ be arbitrary but fixed and take $\epsilon _1$ small enough to ensure $\frac{1}{b-\epsilon _1}\le \frac{1}{c_1}\Leftrightarrow c_1\le b-\epsilon _1$. Note that the choice of $\epsilon _1$ is only depending on chosen $c_1$ but not on *j*.

Thus $\Sigma _L(t)<\lceil \frac{j}{c_1}\rceil$ and so $t<\lambda _{\lceil \frac{j}{c_1}\rceil }$. We take $t:=\mu _j$ and get $\mu _j<\lambda _{\lceil \frac{j}{c_1}\rceil }$, i.e. the second half of (4.11) for all $j\ge j_{\epsilon _1}$ (since $\mu _j<\mu _{j+1}$ for all *j*) with $d:=0$.

Similarly, the second estimate in (4.13) gives $\lfloor \frac{j}{b+\epsilon }\rfloor \le \frac{j}{b+\epsilon }<\Sigma _L(t)$ and so $t\ge \lambda _{\lfloor \frac{j}{b+\epsilon }\rfloor }$. We let $c_2>b$ be arbitrary but fixed and choose $\epsilon _2>0$ small enough to ensure $\frac{1}{b+\epsilon _2}\ge \frac{1}{c_2}\Leftrightarrow c_2\ge b+\epsilon _2$ and so $\lfloor \frac{j}{b+\epsilon _2}\rfloor \ge \lceil \frac{j}{b+\epsilon _2}\rceil -1\ge \lceil \frac{j}{c_2}\rceil -1$. Take $t:=\mu _j$ to get the first half of (4.11) for all $j\ge j_{\epsilon _2}$ with $d:=1$. Summarizing, we are done when taking $j_0:=\max \{j_{\epsilon _1}, j_{\epsilon _2}\}$ and note that *d* can be taken uniformly and not depending on chosen $c_1,c_2$.

$(ii)\Rightarrow (iii)$ This is clear.

$(iii)\Rightarrow (i)$ Let $b\in (0,+\infty )$ be given and take arbitrary (close) $0<c_1<b<c_2$ but from now on fixed. Let $t\ge \mu _{j_0}$ and so $\mu _j\le t<\mu _{j+1}$ for some $j\ge j_0$. Then (4.12) gives

$$\begin{aligned} \lambda _{\lceil j/c_2\rceil -d_j}\le \mu _j\le t<\mu _{j+1}<\lambda _{\lceil (j+1)/c_1\rceil +d_{j+1}}. \end{aligned}$$

So $\Sigma _M(t)=j$ and the first estimate implies $\Sigma _L(t)\ge \lceil \frac{j}{c_2}\rceil -d_j\ge \frac{j}{c_2}-d_j$, whereas the last estimate yields $\Sigma _L(t)\le \lceil \frac{j+1}{c_2}\rceil +d_{j+1}-1\le \frac{j+1}{c_1}+d_{j+1}$. Summarizing, for all such *t* we obtain

$$\begin{aligned} \frac{j}{j/c_1+1/c_1+d_{j+1}}\le \frac{\Sigma _M(t)}{\Sigma _L(t)}\le \frac{j}{j/c_2-d_j}. \end{aligned}$$

Then note that $\frac{d_{j+1}}{j}=\frac{d_{j+1}}{j+1}\frac{j+1}{j}\rightarrow 0$ as $j\rightarrow +\infty \Leftrightarrow t\rightarrow +\infty$. Hence, as $c_1,c_2\rightarrow b$ we get that $\frac{\Sigma _M(t)}{\Sigma _L(t)}\rightarrow b$ as $t\rightarrow +\infty$. $\square$

## From N-functions to associated weight sequences

The aim is to reverse the construction from Sect. 3.2; i.e. we start with an abstractly given N-function *G*, associate to it a sequence $M^G$ and study then the relation between *G* and the associated N-function $F_{M^G}$.

Let *F* be an N-function and first we introduce

5.1 $$\begin{aligned} \omega _F(t):=F(\log (t)),\;\;\;t\ge 1,\hspace{15pt}\omega _F(t):=0,\;\;\;0\le t<1\;\;\;\text {(normalization)}.\nonumber \\ \end{aligned}$$

By the properties of *F* we have that $\omega _F$ belongs to the class $\mathcal {W}_0$: Concerning $(\omega _4)$ note that $\varphi _{\omega _F}(t)=\omega _F(e^t)=F(t)$ for all $t\ge 0$, concerning $(\omega _3)$ we remark that $\frac{\omega _F(t)}{\log (t)}=\frac{\omega _F(e^s)}{s}=\frac{F(s)}{s}$ for all $t>1\Leftrightarrow s>0$ and the last quotient tends to $+\infty$ as $s\rightarrow +\infty$, see (3.3).

*Note* When given $\omega \in {\mathcal {W}_0}$, then one can put

5.2 $$\begin{aligned} F_{\omega }(t):=\varphi _{\omega }(|t|)=\omega (e^{|t|}),\;\;\;t\in \mathbb {R}. \end{aligned}$$

$F_{\omega }$ satisfies all properties to be formally an N-function (see Definition 3.1) except necessarily the first part in (3.3) and also $F_{\omega }(t)>0$ for all $t\ne 0$ is not clear. Thus the set of all *N*-functions does not coincide with the class $\mathcal {W}_0$. However, one can overcome this technical problem for $F_{\omega }$ by passing to an equivalent (associated) N-function when taking into account Theorem 3.9 analogously as it has been done before with $\varphi _{\omega _M}$.

We consider the so-called *Legendre–Fenchel–Young-conjugate* of $\varphi _{\omega _F}$ which is given by

5.3 $$\begin{aligned} \varphi ^{*}_{\omega _F}(s):=\sup \{|s|t-\varphi _{\omega _F}(t): t\ge 0\}=\sup \{|s|t-F(t): t\ge 0\},\;\;\;s\in \mathbb {R}.\nonumber \\ \end{aligned}$$

$\varphi _{\omega _F}$ is non-decreasing, convex by assumption, $\varphi _{\omega _F}(0)=\omega _F(e^0)=F(0)=0$ and $\lim _{t\rightarrow +\infty }\frac{\varphi _{\omega _F}(t)}{t}=\lim _{s\rightarrow +\infty }\frac{\omega _F(s)}{\log (s)}=+\infty$ as seen before. Thus we get, see e.g. [2, Rem. 1.3]:

$$\varphi ^{*}_{\omega _F}$$ is convex, $$\varphi ^{*}_{\omega _F}(0)=0$$, $$s\mapsto \frac{\varphi ^{*}_{\omega _F}(s)}{s}$$ is non-decreasing and tending to $$+\infty $$ as $$s\rightarrow +\infty $$. Finally $$\varphi ^{**}_{\omega _F}(s)=\varphi _{\omega _F}(s)$$ holds for all $$s\ge 0$$.

Next let us introduce the associated sequence $M^F=(M^F_j)_j$ by

5.4 $$\begin{aligned} M^F_j:=\sup _{t>0}\frac{t^j}{\exp (\omega _F(t))}=\sup _{t\ge 1}\frac{t^j}{\exp (\omega _F(t))}=\sup _{t\ge 1}\frac{t^j}{\exp (F(\log (t)))},\;\;\;j\in \mathbb {N}.\nonumber \\ \end{aligned}$$

The second equality holds by normalization and this formula should be compared with (2.5). Hence for any $j\in \mathbb {N}$ we get

$$\begin{aligned} M^F_j&=\sup _{t>0}\frac{t^j}{\exp (\omega _F(t))}=\exp \sup _{t>0}\left( j\log (t)-\omega _F(t)\right) \\&=\exp \sup _{s\in \mathbb {R}}\left( js-\omega _F(e^s)\right) =\exp \sup _{s\ge 0}\left( js-\omega _F(e^s)\right) \\&=\exp \sup _{s\ge 0}\left( js-\varphi _{\omega _F}(s)\right) =\exp \big (\varphi ^{*}_{\omega _F}(j)\big ), \end{aligned}$$

which implies $M^F\in {\mathcal{L}\mathcal{C}}$ by the properties of the conjugate. Note that by definition (normalization) one has $\omega _F(e^s)=0$ for all $-\infty <s\le 0$.

Finally, by [18, Thm. 4.0.3] applied to $\omega =\omega _F$, see also [15, Lemma 5.7] and [8, Lemma 2.5] with weight matrix parameter $x=1$ there, we obtain

5.5 $$\begin{aligned} \exists \;C\ge 1\;\forall \;t\ge 0:\;\;\;\omega _{M^F}(t)\le \omega _F(t)\le 2\omega _{M^F}(t)+C, \end{aligned}$$

hence by (5.1)

5.6 $$\begin{aligned} \exists \;C\ge 1\;\forall \;t\ge 0:\;\;\;\varphi _{\omega _{M^F}}(t)\le \omega _F(e^t)=F(t)\le 2\varphi _{\omega _{M^F}}(t)+C. \end{aligned}$$

In order to avoid confusion let from now in this context *G* be the given N-function, $M^G$ the sequence defined in (5.4) and $F_{M^G}$ the associated N-function from Corollary 3.12 (applied to the sequence $M^G$).

### Definition 5.1

Let *G* be an N-function. Then the sequence $M^G$ is called the associated weight sequence.

### Theorem 5.2

Let *G* be an N-functions and let $F_{M^G}$ be the N-function associated with the sequence $M^G$. Then we get

5.7 $$\begin{aligned} \exists \;A,B\ge 1\;\forall \;t\ge 0:\;\;\;F_{M^G}(t)\le G(t)+A\le 2F_{M^G}(t)+B\le F_{M^G}(2t)+B,\nonumber \\ \end{aligned}$$

and this implies both $G\sim F_{M^G}\sim \varphi _{\omega _{M^G}}$ and $G{\sim _{\mathfrak {c}}}F_{M^G}{\sim _{\mathfrak {c}}}\varphi _{\omega _{M^G}}$.

### Proof

Corollary 3.12 applied to $M^G$ and (5.6) yield

$$\begin{aligned} \exists \;C,C_1\ge 1\;\forall \;t\ge 0:\;\;\;G(t)\le 2\varphi _{\omega _{M^G}}(t)+C\le 2F_{M^G}(t)+C+C_1, \end{aligned}$$

and

$$\begin{aligned} \exists \;C_2\ge 1\;\forall \;t\ge 0:\;\;\;F_{M^G}(t)\le \varphi _{\omega _{M^G}}(t)+C_2\le G(t)+C_2. \end{aligned}$$

These estimates prove the first two parts in (5.7) and the last one there follows by (3.2), so by convexity and normalization of $F_{M^G}$, see also the estimate in (*i*) in Remark 3.5 applied to $a:=2$.

The desired relations follow now by (5.7), Lemma 3.3 and Corollary 3.12. $\square$

The next result gives the (expected) equivalence when starting with a weight sequence.

### Proposition 5.3

Let $L\in {\mathcal{L}\mathcal{C}}$ be given and let $F_L$ be the associated N-function. Then we get

$$\begin{aligned} \exists \;A,B\ge 1\;\forall \;j\in \mathbb {N}:\;\;\;\frac{1}{A}L_j\le M^{F_L}_j\le BL_j. \end{aligned}$$

This estimate implies $M^{F_L}{\approx }L$, $F_L{\sim _{\mathfrak {c}}}F_{M^{F_L}}$ and $F_L\sim F_{M^{F_L}}$.

### Proof

We apply the previous constructions to the associated N-function $F_L$. For all $t\ge 1$ we have $\omega _{F_L}(t)=F_L(\log (t))$ and so by (3.18)

5.8 $$\begin{aligned} \exists \;C,D\ge & {} 1\;\forall \;t\ge 1:\;\;\;\omega _L(t)-C=\varphi _{\omega _L}(\log (t))-C\le \omega _{F_L}(t)\nonumber \\\le & {} \varphi _{\omega _L}(\log (t))+D=\omega _L(t)+D. \end{aligned}$$

Because $L\in {\mathcal{L}\mathcal{C}}$ we have $\omega _L(t)=0$ for all $t\in [0,1]$ (normalization) and $\omega _{F_L}(t)=0$ holds for all $t\in [0,1]$ by (5.1). Thus (5.8) is valid for any $t\ge 0$. By combining (5.8) with (5.4) and (2.5) we arrive at

$$\begin{aligned} \exists \;C,D\ge 1\;\forall \;j\in \mathbb {N}:\;\;\;e^{-D}L_j\le M^{F_L}_j\le e^CL_j, \end{aligned}$$

hence the conclusion.

This relation clearly implies $M^{F_L}{\approx }L$ and so, by Theorem 4.4 (recall also Remark 4.3) the equivalence $F_L{\sim _{\mathfrak {c}}}F_{M^{F_L}}$. Moreover, (4.3) is verified with $c=1$ (pair-wise) and hence Theorem 4.2 gives that $F_L\sim F_{M^{F_L}}$ as well. $\square$

We continue with the following observations:

- Theorem 5.2 suggests that for abstractly given N-functions *G* it is important to have information about $F_{M^G}$ resp. $\varphi _{\omega _{M^G}}$ and to study the associated weight sequence $M^G$. In particular, in view of (3.14) and (3.19) the knowledge about the counting function $\Sigma _{M^G}$ is useful and this amounts to study the sequence of quotients $\mu ^G$.
- On the other hand, when desired growth behaviours are expressed in terms of $\mu ^G$, it is an advantage not to compute first $M^G$ via given *G* and then the corresponding quotient sequence $\mu ^G$ but to come up with a property for *G* directly.
- This can be achieved by relating $\mu ^G$ directly to *G* as follows: Put
  5.9 $$\begin{aligned} v_G(t):=\exp (-G(\log (t))),\;\;\;t\ge 1,\hspace{15pt}v_G(t):=1,\;\;\;0\le t<1, \end{aligned}$$
  and so (5.4) takes alternatively the following form:
  $$\begin{aligned} M^G_j=\sup _{t>0}t^jv_G(t),\;\;\;j\in \mathbb {N}. \end{aligned}$$
  This should be compared with [19, Sect. 6], [22, Sect. 2.6, 2.7]. Indeed, $M^G$ coincides with the crucial associated sequence $M^{v_G}$ in [19, 22]. By the assumptions on *G* we get that $v_G$ is a (normalized and convex) weight in the notion of [19, 22], i.e. in the setting of weighted spaces of entire functions, see also the literature citations in these papers.
- Let now $(t_j)_{j\in \mathbb {N}}$ be the sequence such that $t_j\ge 0$ is denoting a/the global maximum point of the mapping $t\mapsto t^jv_G(t)$.
- The advantage when considering $v_G$ is that in [19, (6.5)] we have derived the following relation between the sequence of quotients $\mu ^G$ (set $\mu ^G_0:=1$) and $(t_j)_{j\in \mathbb {N}}$:
  5.10 $$\begin{aligned} \forall \;j\in \mathbb {N}:\;\;\;t_j\le \mu ^G_{j+1}\le t_{j+1}. \end{aligned}$$
  *Note:* For $j=0$ we have put $t_0:=1$ and so equality with $\mu ^G_0$. In fact for $j=0$ we can choose any $t\in [0,1]$ as $t_0$ since $v_G$ is non-increasing and normalized, and by the convention $0^0:=1$. So $t_j\ge 1$ for any $j\in \mathbb {N}$ because $j\mapsto t_j$ is non-decreasing and since $\lim _{j\rightarrow +\infty }\mu ^G_j=+\infty$ we also get $t_j\rightarrow +\infty$. For concrete given *G* (belonging to $\mathcal {C}^1$) the concrete computation for the values of $t_j$ might be not too difficult.

Then put

5.11 $$\begin{aligned} \Sigma ^G(t):=|\{j\in \mathbb {N}_{>0}:\;\;\;t_j\le t\}|,\;\;\;t\ge 0, \end{aligned}$$

and $\Sigma ^G:[0,+\infty )\rightarrow \mathbb {N}$ is a right-continuous non-decreasing function with $\Sigma ^G(t)=0$ for all $0\le t<1$ and $\Sigma ^G(t)\rightarrow +\infty$ as $t\rightarrow +\infty$. By taking into account (5.10) and the definition of $\Sigma _{M^G}$ and $\Sigma ^G$ we get:

### Proposition 5.4

Let *G* be an N-function and let $M^G$ be the associated weight sequence. Then the counting functions $\Sigma ^G$ and $\Sigma _{M^G}$ are related by

5.12 $$\begin{aligned} \forall \;t\ge 0:\;\;\;\Sigma _{M^G}(t)\le \Sigma ^G(t)+1\le \Sigma _{M^G}(t)+1, \end{aligned}$$

which yields

$$\begin{aligned} \lim _{t\rightarrow +\infty }\frac{\Sigma _{M^G}(t)}{\Sigma ^G(t)}=1. \end{aligned}$$

Using $\Sigma ^G$ we introduce

5.13 $$\begin{aligned} \varphi ^G(x):=\int _0^{|x|}\Sigma ^G(e^t)dt,\;\;\;x\in \mathbb {R}. \end{aligned}$$

Note that, analogously to the explanations for $\varphi _{\omega _M}$ given in Remark 3.7 in Sect. 3.2 we have that $\varphi ^G$ is formally not an N-function since $t_j\ge 1$ for all $j\ge 1$ and so by definition $\Sigma ^G(e^0)\ge 1\ne 0$ if $t_1=1$ or $\Sigma ^G(e^t)=0$ for all $0\le t<\log (t_1)$ if $t_1>1$. We summarize the whole information in the final statement of this section:

### Theorem 5.5

Let *G* be an N-function, $M^G$ the associated weight sequence, $F_{M^G}$ the associated N-function and $\varphi ^G$ given by (5.13). Then

$$\begin{aligned} G{\sim _{\mathfrak {c}}}F_{M^G}{\sim _{\mathfrak {c}}}\varphi _{\omega _{M^G}}{\sim _{\mathfrak {c}}}\varphi ^G,\hspace{15pt}G\sim F_{M^G}\sim \varphi _{\omega _{M^G}}\sim \varphi ^G. \end{aligned}$$

### Proof

The first two parts are shown in Theorem 5.2. The last one follows by Proposition 5.4: We use (5.12) and the representations (5.13) and (3.14) in order to get:

5.14 $$\begin{aligned} \exists \;C\ge 1\;\forall \;t\ge 0:\;\;\;\varphi ^G(t)\le \varphi _{\omega _{M^G}}(t)\le \varphi ^G(t)+t\le 2\varphi ^G(t)+C\le \varphi ^G(2t)+C.\nonumber \\ \end{aligned}$$

The second last estimate in (5.14) holds since $\lim _{t\rightarrow +\infty }\frac{\varphi ^G(t)}{t}=+\infty$ and the last one by (3.2) applied to $\varphi ^G$. Both requirements on $\varphi ^G$ are valid by the representation (5.13), see Remark 3.11. Then (5.14) implies both $\varphi _{\omega _{M^G}}{\sim _{\mathfrak {c}}}\varphi ^G$ and $\varphi _{\omega _{M^G}}\sim \varphi ^G$. $\square$

Theorem 5.5 shows that, up to equivalence, the whole information concerning growth and regularity properties of *G* is already expressed by involving a certain associated weight sequence $M^G$ and its related/associated N-function $F_{M^G}$.

## On complementary N-functions and dual sequences

We give a connection between the so-called complementary N-functions $F^c$ and the dual sequences *D* in the weight sequence setting. Note that in the theory of N-functions one naturally has the pair $(F,F^c$) and thus also $(F_M,F_M^c)$. Similarly for each $M\in {\mathcal{L}\mathcal{C}}$ one can naturally assign the dual sequence $D\in {\mathcal{L}\mathcal{C}}$ and we show that $F_M^c$ is closely related to *D* via an integral representation formula using the counting-function $\log \circ \Sigma _D$.

### Complementary N-functions in the weight sequence setting

Let *F* be an N-function given by the representation (3.1) with right-derivative *f*. First, introduce

6.1 $$\begin{aligned} f^c(s):=\sup \{t\ge 0: f(t)\le s\},\;\;\;s\ge 0, \end{aligned}$$

thus $f^c$ is the right-inverse of *f*, i.e. it is the right-inverse of the right-derivative of *F*, see [11, (2.1)]. It is known that $f^c$ satisfies (I)–(III) in Sect. 3.1. (6.1) should be compared with [17, (13), p. 10]; the difference appears since in [17] the authors work with the integral representation involving the left-continuous integrand/density (see Remark 3.2).

#### Definition 6.1

The *complementary N-function* $F^c$, see [11, Chapter I, §2, p. 11], is defined by

6.2 $$\begin{aligned} F^c(x):=\int _0^{|x|}f^c(t)dt,\;\;\;x\in \mathbb {R}. \end{aligned}$$

In [11, Chapter I, (2.9), p. 13] it is mentioned that

6.3 $$\begin{aligned} F^c(s)=\max _{t\ge 0}\{|s|t-F(t)\},\;\;\;s\in \mathbb {R}, \end{aligned}$$

and this formula can be considered as an equivalent definition for $F^c$.

#### Remark 6.2

We comment on the comparison of growth relations $\preceq _{\mathfrak {c}}$ and $\preceq$ between (associated) N-functions *F*, *G* and their complementary N-functions $F^c$, $G^c$.

(i) In [11, Thm. 3.1, Thm. 3.2] it has been shown that $F{\preceq _{\mathfrak {c}}}G$ implies $G^c{\preceq _{\mathfrak {c}}}F^c$ and by [17, Sect. 2.2, Thm. 2 (*a*)] in fact this is an equivalence. By [17, Sect. 2.2, Thm. 2 (*b*)] the corresponding statements is valid w.r.t. relation $\preceq \preceq _{\mathfrak {c}}$ as well. In particular, two N-functions are equivalent if and only if their complementary N-functions so are.
(ii) If $F\preceq G$, then $G(t)\le CF(t)+D$ for some $C,D\ge 1$ and all $t\ge 0$ and so (6.3) gives for any $s\in \mathbb {R}$
  $$\begin{aligned} G^c(s)&=\max _{t\ge 0}\{|s|t-G(t)\}\ge \max _{t\ge 0}\{|s|t-CF(t)\}-D\\&=C\max _{t\ge 0}\{C^{-1}|s|t-F(t)\}-D\\&=CF^c(s/C)-D, \end{aligned}$$
  see also the proof of [15, Lemma 5.16]. This relation implies (3.7) and so Lemma 3.3 yields that $F^c{\preceq _{\mathfrak {c}}}G^c$. When one can choose $C=1$, then also $G^c\preceq F^c$ follows but in general this implication is not clear; see also Lemma 7.1.

By combining (5.3) and (6.3) we get

$$\begin{aligned} \varphi ^{*}_{\omega _F}=F^c, \end{aligned}$$

and since $M^F_j=\exp (\varphi ^{*}_{\omega _F}(j))$, for this see (5.4) and the computations below this equation, it follows that

$$\begin{aligned} \forall \;j\in \mathbb {N}:\;\;\;M^F_j=\exp (F^c(j)). \end{aligned}$$

Let now $M\in {\mathcal{L}\mathcal{C}}$ be given, then write $F^c_M$ and $f^c_M$ for the functions considered before w.r.t. to the associated N-function $F_M$ and the corresponding right-derivative $f_M$. Moreover, in view of (6.1), let us introduce

6.4 $$\begin{aligned} \Gamma _M(s):=\sup \{t\ge 0: \Sigma _M(e^t)\le s\},\;\;\;s\ge 0. \end{aligned}$$

Finally we set

6.5 $$\begin{aligned} \varphi ^c_{\omega _M}(x):=\int _0^{|x|}\Gamma _M(s)ds,\;\;\;x\in \mathbb {R}. \end{aligned}$$

By (3.19) it follows that

6.6 $$\begin{aligned} \exists \;s_0>0\;\forall \;s\ge s_0:\;\;\;f^c_M(s)=\Gamma _M(s), \end{aligned}$$

because both functions are non-decreasing and $f_M(t)=\Sigma _M(e^t)$ for all $t\ge t_0$, with $t_0$ the value appearing in (3.19). So (6.6) is valid for all $s\ge s_0:=f_M(t_0)$. Using this identity we can prove the analogous result of Corollary 3.12 for the functions $F^c_M$ and $\varphi ^c_{\omega _M}$.

#### Proposition 6.3

Let $M\in {\mathcal{L}\mathcal{C}}$ be given. Then we get

$$\begin{aligned} \exists \;C,D\ge 1\;\forall \;t\ge 0:\;\;\;\varphi ^c_{\omega _M}(t)-C\le F^c_M(t)\le \varphi ^c_{\omega _M}(t)+D. \end{aligned}$$

This implies that both $\varphi ^c_{\omega _M}{\sim _{\mathfrak {c}}}F^c_M$ and $\varphi ^c_{\omega _M}\sim F^c_M$ hold.

#### Proof

We use (6.6) and the representations (6.2) and (6.5) (recall also the arguments in the proof of Proposition 3.10). For all $s\ge s_0$ (with $s_0$ from (6.6)) we get

$$\begin{aligned} \varphi ^c_{\omega _M}(s)&=\int _0^s\Gamma _M(t)dt=\int _0^{s_0}\Gamma _M(t)dt+\int _{s_0}^s\Gamma _M(t)dt\\&=\int _0^{s_0}\Gamma _M(t)dt +\int _{s_0}^sf^c_M(t)dt\\&\le \int _0^{s_0}\Gamma _M(t)dt+\int _0^sf_M(t)dt=\varphi ^c_{\omega _M}(s_0)+F_M^c(s), \end{aligned}$$

hence $\varphi ^c_{\omega _M}(s)\le F_M^c(s)+C$ for all $s\ge 0$ when choosing $C:=\varphi ^c_{\omega _M}(s_0)$. Similarly, for all $s\ge s_0$

$$\begin{aligned} F^c_M(s)&=\int _0^sf_M^c(t)dt=\int _0^{s_0}f_M^c(t)dt+\int _{s_0}^sf_M^c(t)dt=\int _0^{s_0}f_M^c(t)dt+\int _{s_0}^s\Gamma _M(t)dt \\ {}&\le \int _0^{s_0}f_M^c(t)dt+\int _0^s\Gamma _M(t)dt=F^c_M(s_0)+\varphi ^c_{\omega _M}(s), \end{aligned}$$

hence $F^c_M(s)\le \varphi ^c_{\omega _M}(s)+D$ for all $s\ge 0$ when choosing $D:=F^c_M(s_0)$. $\square$

On the other hand, formula (6.3) applied to $\varphi _{\omega _M}$ yields

6.7 $$\begin{aligned} \sup _{t\ge 0}\{|s|t-\varphi _{\omega _M}(t)\}=\varphi ^{*}_{\omega _M}(s),\;\;\;s\in \mathbb {R}, \end{aligned}$$

the *Legendre–Fenchel–Young-conjugate* of $\varphi _{\omega _M}$. By combining (3.18), (6.3) applied to $F_M$ and (6.7) we get

6.8 $$\begin{aligned} \exists \;C,D\ge 1\;\forall \;s\in \mathbb {R}:\;\;\;-C+F^c_M(s)\le \varphi ^{*}_{\omega _M}(s)\le F^c_M(s)+D, \end{aligned}$$

see the estimate in (*ii*) in Remark 6.2. Hence $F^c_M{\sim _{\mathfrak {c}}}\varphi ^{*}_{\omega _M}$ and $F^c_M\sim \varphi ^{*}_{\omega _M}$ hold. When combining this with Proposition 6.3 we arrive at the following result:

#### Theorem 6.4

Let $M\in {\mathcal{L}\mathcal{C}}$ be given. Then

$$\begin{aligned} \varphi ^c_{\omega _M}{\sim _{\mathfrak {c}}}F^c_M{\sim _{\mathfrak {c}}}\varphi ^{*}_{\omega _M},\hspace{15pt}\varphi ^c_{\omega _M}\sim F^c_M\sim \varphi ^{*}_{\omega _M}. \end{aligned}$$

Finally, we apply Theorem 6.4 to the associated weight sequence $M^G$.

#### Corollary 6.5

Let *G* be an N-function, let $M^G\in {\mathcal{L}\mathcal{C}}$ be the associated sequence defined via (5.4) and $\varphi ^G$ be given by (5.13). Then

$$\begin{aligned} \varphi ^{*}_{\omega _{M^G}}{\sim _{\mathfrak {c}}}\varphi ^c_{\omega _{M^G}}{\sim _{\mathfrak {c}}}F^c_{M^G}{\sim _{\mathfrak {c}}}G^c{\sim _{\mathfrak {c}}}(\varphi ^G)^c,\hspace{15pt}\varphi ^{*}_{\omega _{M^G}}\sim \varphi ^c_{\omega _{M^G}}\sim F^c_{M^G}. \end{aligned}$$

#### Proof

Concerning $\sim _{\mathfrak {c}}$, the first and the second equivalence hold by Theorem 6.4, the third and the fourth one by taking into account (5.7), Theorem 5.5 (see (5.14)) and (*i*) in Remark 6.2. Here $(\varphi ^G)^c$ is given in terms of (6.3).

For $\sim$ we use the same results, however $F^c_{M^G}\sim G^c$ and $G^c\sim (\varphi ^G)^c$ are not clear in general: In order to conclude we want to apply (6.3) and so in the relations only an additive constant should appear, see (*ii*) in Remark 6.2. However, in both (5.7) and (5.14) we also have the multiplicative constant 2. $\square$

### Complementary associated N-functions versus dual sequences

In this section the aim is to see that $\Gamma _M$ in (6.4) and crucially appearing in the representation (6.5) is closely connected to the counting function $\Sigma _D$, with *D* denoting the so-called *dual sequence* of *M*. Moreover, (6.4) should be compared with the formula for the so-called *bidual sequence* of *M* in [5, Def. 2.1.41, Thm. 2.1.42].

For given $M\in {\mathcal{L}\mathcal{C}}$ we introduce the *dual sequence* $D=(D_j)_j$ defined in terms of the corresponding quotient sequence $\delta$ as follows, see [5, Def. 2.1.40, p. 81]:

6.9 $$\begin{aligned} \forall \;j\ge \mu _1(\ge 1):\;\;\;\delta _{j+1}:=\Sigma _M(j),\hspace{20pt}\delta _{j+1}:=1\;\;\;\forall \;j\in \mathbb {Z},\;-1\le j<\mu _1.\nonumber \\ \end{aligned}$$

Then we set

$$\begin{aligned} D_j:=\prod _{k=0}^j\delta _k,\;\;\;j\in \mathbb {N}, \end{aligned}$$

hence $D\in {\mathcal{L}\mathcal{C}}$ with $1=D_0=D_1$ follows. Recall that by [5, Def. 2.1.27] the function $\nu _{\textbf{m}}$ in [5] precisely denotes the counting function $\Sigma _M$ (see (2.2)) and note that concerning the sequence of quotients there exists an index-shift: more precisely we have $m_j\equiv \mu _{j+1}$ for all $j\in \mathbb {N}$ with $\textbf{m}=(m_j)_j$ used in [5] and [7]. Moreover, a weight sequence in [5] means a sequence having all requirements from class $\mathcal{L}\mathcal{C}$ except $M_0\le M_1$; see [5, Sect. 1.1.1, Def. 1.1.8]. Finally, we mention that this notion should be compared with the definition of the counting function $\varphi$ on [17, Sect. 1.2, p. 3].

Let $M\in {\mathcal{L}\mathcal{C}}$ be given, we analyze now $\Gamma _M$:

- Obviously, $\lim _{s\rightarrow +\infty }\Gamma _M(s)=+\infty$ and since $\lim _{j\rightarrow +\infty }\mu _j=+\infty$ we have $\mu _j<\mu _{j+1}$ for infinitely many indices *j*.
- Then recall that $\Sigma _M(e^t)\in \mathbb {N}$ and that $\Sigma _M(e^t)=0$ for all $0\le t<\log (\mu _1)$. By normalization we get $\log (\mu _j)\ge \log (\mu _1)\ge \log (1)=0$ for all $j\in \mathbb {N}_{>0}$.
- *Case I:* Assume that $1=\mu _1=\dots =\mu _d<\mu _{d+1}$ for some $d\in \mathbb {N}_{>0}$. Then $e^t\ge \mu _d=1$ and so $\Sigma _M(e^t)\ge d$ for all $t\ge 0$. Hence the set of values $t\ge 0$ with $\Sigma _M(e^t)\le s$ in the definition of $\Gamma _M(s)$ is empty for all values *s* with $0\le s<d$. In this case we put $\Gamma _M(s):=0$. Note that *d* is finite because $\lim _{j\rightarrow +\infty }\mu _j=+\infty$. Let now *s* be such that $j\le s<j+1$ for some $j\in \mathbb {N}_{>0}$ with $j\ge d$. In view of Remark 2.2 in general it is not clear that we can find $t\ge 0$ such that $\Sigma _M(e^t)=j$. In fact this holds if and only if $\mu _j<\mu _{j+1}$ because for all $t\ge 0$ with $\log (\mu _j)\le t<\log (\mu _{j+1})$ we get $\Sigma _M(e^t)=j$. We have $\Sigma (e^{\log (\mu _{j+1})})\ge j+1>s$ and consequently for all such indices *j*, in particular for $j=d$, we obtain $\Gamma _M(s)=\log (\mu _{j+1})$. If $j\ge d$ is such that $\mu _j=\mu _{j+1}$, then $j\ge d+1$. Thus there exist $\ell ,c\in \mathbb {N}_{>0}$ with $d\le \ell \le j-1$ and such that $\mu _{\ell }<\mu _{\ell +1}=\mu _{\ell +2}=\dots =\mu _{\ell +c}=\mu _j=\mu _{j+1}$. For all *t* with $\log (\mu _{\ell })\le t<\log (\mu _{\ell +1})$ we get $\Sigma _M(e^t)=\ell <j$ and since $\Sigma _M(e^{\log (\mu _{\ell +1})})=\Sigma _M(e^{\log (\mu _{j+1})})\ge j+1>s$ we have again $\Gamma _M(s)=\log (\mu _{j+1})$.
- *Case II:* Assume that $\mu _1>1$. First we have $\Gamma _M(s)=\log (\mu _1)(>0)$ for all $0\le s<1$ since $\Sigma _M(e^t)=0$ for all $0\le t<\log (\mu _1)$ and $\Sigma _M(e^{\log (\mu _1)})\ge 1$. Let now $j\le s<j+1$ for some $j\in \mathbb {N}_{>0}$. Similarly as above, if $\mu _j<\mu _{j+1}$ then we get $\Gamma _M(s)=\log (\mu _{j+1})$. If $\mu _j=\mu _{j+1}$, then we distinguish: Either there exist $\ell ,c\in \mathbb {N}_{>0}$ with $1\le \ell \le j-1$ such that $\mu _{\ell }<\mu _{\ell +1}=\mu _{\ell +2}=\dots =\mu _{\ell +c}=\mu _j=\mu _{j+1}$. Then again $\Gamma _M(s)=\log (\mu _{j+1})$ by the same reasons as in Case I before. Finally, if this choice is not possible, then this precisely means $\mu _1=\cdots =\mu _j=\mu _{j+1}$. Since $\mu _1>1$ we have $\Sigma _M(e^t)=0$ for all $0\le t<\log (\mu _1)$ and $\Sigma _M(e^t)\ge j+1>s$ for all $t\ge \log (\mu _{j+1})=\log (\mu _1)$. Thus $\Gamma _M(s)=\log (\mu _{j+1})=\log (\mu _1)$ holds.

Summarizing, we have shown the following statement:

#### Proposition 6.6

Let $M\in {\mathcal{L}\mathcal{C}}$ be given with quotient sequence $(\mu _j)_j$.

- If $1=\mu _1=\dots =\mu _d<\mu _{d+1}$ for some $d\in \mathbb {N}_{>0}$, then
  $$\begin{aligned} \Gamma _M(s)=0,\;\;\;\forall \;0\le s<d,\hspace{25pt}\Gamma _M(s)=\log (\mu _{j+1}),\;\;\;\;\forall \;j\le s<j+1,\;\forall \;j\ge d. \end{aligned}$$
- If $$\mu _1>1$$, then
  $$\begin{aligned} \Gamma _M(s)=\log (\mu _{j+1}),\;\;\;\forall \;j\le s<j+1,\;\forall \;j\in \mathbb {N}. \end{aligned}$$

Next let us study $\Sigma _D$ in detail, see also the proof of [5, Thm. 2.1.42]:

- Let $t\ge 0$, then the value $\Sigma _D(t)$ is equal to the maximal integer $j\in \mathbb {N}_{>0}$ such that $\delta _j\le t$, if such *j* exists, and otherwise equal to 0. By definition in (6.9) we have $\delta _j\in \mathbb {N}_{>0}$ for all $j\in \mathbb {N}$ and so $\Sigma _D(t)=0$ for $0\le t<1$.
- In fact $\delta _j=1$ for all $j\in \mathbb {N}_{>0}$ such that $j<\mu _1+1$. The largest of those integers *j* coincides with $\lfloor \mu _1+1\rfloor \ge 2$ if $\mu _1\notin \mathbb {N}_{>0}$ (in this case $\mu _1>1$) and with $\mu _1$ if $\mu _1\in \mathbb {N}_{>0}$. Moreover, $\delta _j=1$ for all *j* such that $\mu _1+1\le j<\mu _2+1$ if such an integer *j* exists (case I). In particular, this holds if $\mu _2\ge \mu _1+1$. If not, then $\delta _j=d\in \mathbb {N}_{\ge 2}$ for the minimal integer *j* with $j\ge \mu _1+1$ (case II). This occurs if for this minimal integer already $j\ge \mu _d+1$ is valid. Note that *d* is finite since $\lim _{j\rightarrow +\infty }\mu _j=+\infty$ and let *d* be such that $\mu _d+1\le j<\mu _{d+1}+1$ for *j* minimal such that $j\ge \mu _1+1$.
- Consider $1\le t<2$ and distinguish: In the first case $\Sigma _D(t)$ is the largest integer *j* with $\mu _1+1\le j<\mu _2+1$ and in the second case $\Sigma _D(t)$ coincides with the largest integer *j* with $j<\mu _1+1$. Since $\mu _1+1\ge 2$ in both cases the existence of such an integer *j* is ensured.
- More generally, in case I if $n\le t<n+1$ with $n\in \mathbb {N}_{>0}$, then $\Sigma _D(t)$ is the largest integer *j* such that $j<\mu _{n+1}+1$.
- In case II, if $n\le t<n+1$ for some $n\in \mathbb {N}_{>0}$ with $n+1\le d$, then $\Sigma _D(t)$ is (still) the largest integer *j* such that $j<\mu _1+1$ since for the minimal integer $j\ge \mu _1+1$ we have $\delta _{j}=\Sigma _M(j-1)\ge \Sigma _M(\mu _d)=d>t$. The last equality holds since $\mu _d<\mu _{d+1}$ by the choice of *d*. If $n\le t<n+1$ for some $n\in \mathbb {N}_{>0}$ with $n\ge d$, then $\delta _j$ coincides with largest integer *j* such that $j<\mu _{n+1}+1$. For $n=d$ recall that at least one integer *j* exists with $\mu _d+1\le j<\mu _{d+1}+1$ and so $\delta _j=\Sigma _M(j-1)=d$.

Summarizing, we have shown the following statement (see also [5, Thm. 2.1.42]):

#### Proposition 6.7

Let $M\in {\mathcal{L}\mathcal{C}}$ be given and *D* its dual sequence. Then

$$\begin{aligned} \Sigma _D(t)=0,\;\;\;\forall \;0\le t<1, \end{aligned}$$

and:

- If for the minimal integer *j* such that $j\ge \mu _1+1$ one has $\mu _d+1\le j<\mu _{d+1}+1$ for some $d\in \mathbb {N}_{>0}$, $d\ge 2$, then
  $$\begin{aligned} \Sigma _D(t)= & {} \max \{j\in \mathbb {N}_{>0}:\;j<\mu _1+1\},\;\;\;\forall \;n\in \mathbb {N}_{>0},\;n<d,\;\;\;\forall \;n\le t<n+1,\\ \Sigma _D(t)= & {} \max \{j\in \mathbb {N}_{>0}:\;j<\mu _{n+1}+1\},\;\;\;\forall \;n\in \mathbb {N}_{>0},\;n\ge d,\;\;\;\forall \;n\le t<n+1. \end{aligned}$$
- If for the minimal integer *j* such that $j\ge \mu _1+1$ one has $\mu _1+1\le j<\mu _2+1$, then
  $$\begin{aligned} \Sigma _D(t)=\max \{j\in \mathbb {N}_{>0}:\;j<\mu _{n+1}+1\},\;\;\;\forall \;n\in \mathbb {N}_{>0},\;\;\;\forall \;n\le t<n+1. \end{aligned}$$

When combining Propositions 6.6 and 6.7 we are able to establish a connection between the counting functions $\Gamma _M$ and $\Sigma _D$.

#### Theorem 6.8

Let $M\in {\mathcal{L}\mathcal{C}}$ be given and *D* its dual sequence. Then

6.10 $$\begin{aligned} \exists \;t_0>0\;\forall \;t\ge t_0:\;\;\;\Gamma _M(t)\le \log (\Sigma _D(t))\le \Gamma _M(t)+1, \end{aligned}$$

and this estimate implies

$$\begin{aligned} \lim _{t\rightarrow +\infty }\frac{\Gamma _M(t)}{\log (\Sigma _D(t))}=1. \end{aligned}$$

In (6.10) we can take $t_0:=d\in \mathbb {N}_{>0}$ such that for the minimal integer $j\ge \mu _1+1$ we get $\mu _d+1\le j<\mu _{d+1}+1$.

#### Proof

First, by Proposition 6.7 we get that $\Sigma _D(t)$ coincides with the maximal integer $j<\mu _{n+1}+1$ for all $t\ge 0$ having $n\le t<n+1$ and all $n\ge d$ with $d\in \mathbb {N}_{>0}$ such that for the minimal integer $j\ge \mu _1+1$ we get $\mu _d+1\le j<\mu _{d+1}+1$. In particular, for all such *n* we get $\mu _{n+1}\ge \mu _{d+1}>\mu _1\ge 1$.

Then recall that by Proposition 6.6 we get $\Gamma _M(t)=\log (\mu _{n+1})$ for all *t* such that $n\le t<n+1$ with $n\in \mathbb {N}$ such that $\mu _{n+1}>1$. In particular, as mentioned before, this holds for all $n\ge d$.

So let $n\in \mathbb {N}_{>0}$ be such that $n\ge d$. Take *t* with $n\le t<n+1$, then one has $\Sigma _D(t)<\mu _{n+1}+1$ and

$$\begin{aligned} \log (\Sigma _D(t))<\log (\mu _{n+1}+1)\le \log (e\mu _{n+1})=\log (\mu _{n+1})+1=\Gamma _M(t)+1, \end{aligned}$$

showing the second part of (6.10).

On the other hand for all such *t* we get $\Sigma _D(t)\ge \mu _{n+1}=\exp (\Gamma _M(t))$, in fact even $\Sigma _D(t)\ge \lceil \mu _{n+1}\rceil$ holds, and which proves the first estimate in (6.10). $\square$

Using the counting function $\Sigma _D$ we set

6.11 $$\begin{aligned} F_{\widetilde{\Gamma }_D}(x):=\int _0^{|x|}\widetilde{\Gamma }_D(s)ds,\;\;\;x\in \mathbb {R}, \end{aligned}$$

with

$$\begin{aligned} \widetilde{\Gamma }_D(s):=\log (\Sigma _D(s)),\;\;\;s\ge 1,\hspace{15pt}\widetilde{\Gamma }_D(s):=0,\;\;\;0\le s<1. \end{aligned}$$

$\widetilde{\Gamma }_D$ is non-decreasing and right-continuous and tending to infinity. Recall that $\Sigma _D(s)\in \mathbb {N}_{>0}$ for all $s\ge 1$ and this technical modification is unavoidable: In (6.11) we cannot consider $\log (\Sigma _D(s))$ directly for all $s\ge 0$ because $\Sigma _D(s)=0$ for $0\le s<1$ by definition. Then in view of (6.10) we get

6.12 $$\begin{aligned} \exists \;C,D\ge 1\;\forall \;t\ge 0:\;\;\;-C+\Gamma _M(t)\le \widetilde{\Gamma }_D(t)\le \Gamma _M(t)+D, \end{aligned}$$

which implies

$$\begin{aligned} \lim _{t\rightarrow +\infty }\frac{\Gamma _M(t)}{\widetilde{\Gamma }_D(t)}=1. \end{aligned}$$

Note that $F_{\widetilde{\Gamma }_D}$ is formally not an N-function by analogous reasons as in Remark 3.7: We have $\widetilde{\Gamma }_D(s)=0$ for (at least) all $0\le s<1$, see the proof of Proposition 6.7.

Now we are able to prove the main statement of this section.

#### Theorem 6.9

We have the following equivalences:

(i) Let $M\in {\mathcal{L}\mathcal{C}}$ be given and *D* its dual sequence, then
  $$\begin{aligned} F_{\widetilde{\Gamma }_D}{\sim _{\mathfrak {c}}}\varphi ^c_{\omega _M}{\sim _{\mathfrak {c}}}F^c_M{\sim _{\mathfrak {c}}}\varphi ^{*}_{\omega _M},\hspace{15pt}F_{\widetilde{\Gamma }_D}\sim \varphi ^c_{\omega _M}\sim F^c_M\sim \varphi ^{*}_{\omega _M}. \end{aligned}$$
(ii) Let *G* be an N-function, $M^G\in {\mathcal{L}\mathcal{C}}$ the associated sequence defined via (5.4) and $D^G$ the corresponding dual sequence. Finally, let $\varphi ^G$ be given by (5.13). Then
  $$\begin{aligned} F_{\widetilde{\Gamma }_{D^G}}{\sim _{\mathfrak {c}}}\varphi ^{*}_{\omega _{M^G}}{\sim _{\mathfrak {c}}}\varphi ^c_{\omega _{M^G}}{\sim _{\mathfrak {c}}}F^c_{M^G}{\sim _{\mathfrak {c}}} G^c{\sim _{\mathfrak {c}}}(\varphi ^G)^c,\hspace{15pt}F_{\widetilde{\Gamma }_{D^G}}\sim \varphi ^{*}_{\omega _{M^G}}\sim \varphi ^c_{\omega _{M^G}}\sim F^c_{M^G}. \end{aligned}$$

#### Proof

(*i*) In view of Theorem 6.4 only $F_{\widetilde{\Gamma }_D}{\sim _{\mathfrak {c}}}\varphi ^c_{\omega _M}$ resp. $F_{\widetilde{\Gamma }_D}\sim \varphi ^c_{\omega _M}$ has to be verified. This follows by using (6.12) and the representations (6.11) and (6.5). Note that these representations imply $\lim _{t\rightarrow +\infty }\frac{F_{\widetilde{\Gamma }_D}(t)}{t}=\lim _{t\rightarrow +\infty }\frac{\varphi ^c_{\omega _M}(t)}{t}=+\infty$, see Remark 3.11.

(*ii*) By Corollary 6.5 it suffices to verify $F_{\widetilde{\Gamma }_{D^G}}{\sim _{\mathfrak {c}}}\varphi ^c_{\omega _{M^G}}$ and $F_{\widetilde{\Gamma }_{D^G}}\sim \varphi ^c_{\omega _{M^G}}$. Both relations follow by applying the first part to $M^G$ and $D^G$. Concerning $\sim$ note that here both functions are given directly by the representations (6.11) resp. (6.5), so we are not involving formula (6.3) and since in (6.12) only additive constants appear the problem described in the proof of Corollary 6.5 (see (*ii*) in Remark 6.2) does not occur. $\square$

We remark that $F_{\widetilde{\Gamma }_D}$ does not coincide with $F_D$ directly but nevertheless the dual sequence *D* can be used to get an alternative (equivalent) representation and description of the complementary N-function $F^c_M$.

## Growth and regularity conditions for associated N-functions

In the theory of Orlicz classes and Orlicz spaces several conditions for (abstractly given) N-functions appear frequently. The aim of this section is to study these known growth and regularity assumptions in the weight sequence setting in terms of given *M*.

We remark that all appearing conditions are naturally preserved under $\sim _{\mathfrak {c}}$ for (associated) N-functions, see the given citations in the forthcoming sections resp. Remark 7.6. However, by inspecting the proofs one can see that this fact also holds for a wider class of functions, e.g. when having normalization, convexity, being non-decreasing and tending to infinity (in particular for $\varphi _{\omega _M}$).

Therefore recall that the technical failure of $\varphi _{\omega _M}$ to be formally an N-function occurs at the point 0 (see Remark 3.7) whereas for all crucial conditions under consideration large values $t\ge t_0>0$ are relevant.

Of course, it makes also sense to consider the conditions in this section for arbitrary functions $F:[0,+\infty )\rightarrow [0,+\infty )$.

### The $\Delta _2$-condition

The most prominent property is the so-called $\Delta _2$-condition, see e.g. [11, Chapter I, §4, p. 23] and [17, Sect. 2.3, Def. 1, p. 22], which reads as follows:

7.1 $$\begin{aligned} \limsup _{t\rightarrow +\infty }\frac{F(2t)}{F(t)}<+\infty . \end{aligned}$$

This growth-condition is precisely $(\omega _1)$ for *F* and thus also frequently appears for so-called weight functions $\omega$ in the sense of Braun–Meise–Taylor, see [2]. It is straight-forward that $\Delta _2$ is preserved under relation $\sim$ and also under $\sim _{\mathfrak {c}}$, see [11, p. 23].

It is known, see e.g. [11, Thm. 8.2] and [1, Thm. 3.4], that $\mathcal {L}_F$ is a linear space if and only if *F* satisfies the $\Delta _2$-condition. Moreover, we also get:

#### Lemma 7.1

Let $F_1$ and $F_2$ be two N-functions such that either $F_1$ or $F_2$ has $\Delta _2$. Then $F_1{\preceq _{\mathfrak {c}}}F_2$ if and only if $F_2\preceq F_1$.

In fact, in order to conclude, we only require that either $F_1$ or $F_2$ is normalized and convex and that either $F_1$ or $F_2$ has $\Delta _2$.

#### Proof

By (3.2) we have that $F_1\preceq F_2$ implies $F_2{\preceq _{\mathfrak {c}}}F_1$ (see Remark 3.6). The converse implication holds by an iterated application of $\Delta _2$ for either $F_1$ or $F_2$. $\square$

In the weight sequence setting in view of Corollary 3.12 we require (7.1) (i.e. $(\omega _1)$) not for $\omega _M$ directly but for $\varphi _{\omega _M}$. Note that in [20, Thm. 3.1] we have already given a characterization of $(\omega _1)$ for $\omega _M$ in terms of *M* but $\Delta _2$ for $\varphi _{\omega _M}$ (resp. equivalently for $F_M$) does precisely mean

7.2 $$\begin{aligned} \limsup _{t\rightarrow +\infty }\frac{\omega _M(t^2)}{\omega _M(t)}<+\infty , \end{aligned}$$

which is obviously stronger than $(\omega _1)$ for $\omega _M$. Concerning this requirement we formulate the following result.

#### Theorem 7.2

Let $M\in {\mathcal{L}\mathcal{C}}$ be given. Then the following are equivalent:

(i) The associated N-function $F_M$ (see Corollary 3.12) satisfies the $\Delta _2$-condition.
(ii) $\varphi _{\omega _M}$ satisfies the $\Delta _2$-condition.
(iii) $$\omega _M$$ satisfies (7.2).
(iv) $$\omega _M$$ satisfies
  7.3 $$\begin{aligned} \exists \;C>0\;\exists \;H>0\;\forall \;t\ge 0:\;\;\;\omega _M(t^2)\le C\omega _M(Ht)+C. \end{aligned}$$
(v) *M* satisfies
  7.4 $$\begin{aligned} \exists \;k\in \mathbb {N}_{>0}\;\exists \;A,B\ge 1\;\forall \;j\in \mathbb {N}:\;\;\;(M_j)^{2k}\le AB^jM_{kj}. \end{aligned}$$

(7.3) has already appeared (crucially) in different contexts; it is denoted by $(\omega _7)$ in [9] and in [18]; by $(\omega _8)$ in [15] and by $\Xi$ in [3].

#### Proof

$(i)\Leftrightarrow (ii)$ This is clear by Corollary 3.12 since $F_M{\sim _{\mathfrak {c}}}\varphi _{\omega _M}$.

$(ii)\Leftrightarrow (iii)$ This is immediate.

$(iii)\Rightarrow (iv)$ This is clear since $\omega _M$ is non-decreasing, $\lim _{t\rightarrow +\infty }\omega _M(t)=+\infty$ and w.l.o.g. $H\ge 1$.

$(iv)\Rightarrow (iii)$ As mentioned at the beginning of [9, Appendix A] each non-decreasing function with property (7.3) has already $(\omega _1)$. By iterating this property we get $\omega _M(t^2)\le C_1\omega _M(t)+C_1$ for some $C_1\ge 1$ and all $t\ge 0$, i.e. (7.2).

$(iv)\Leftrightarrow (v)$ This has been shown in [3, Lemma 6.3, Cor. 6.4]. $\square$

In [17, Sect. 2.3, Thm. 3 (*a*)] a characterization of $\Delta _2$ in terms of the left-continuous density/left-derivative of *F* (see Remark 3.2) has been obtained, too.

#### Example 7.3

We list some examples and their consequences:

- According to [3, Lemma 6.5] we know that any $M\in {\mathcal{L}\mathcal{C}}$ cannot satisfy $({\text {mg}})$ and (7.4) simultaneously. In particular, the Gevrey sequences $G^s:=(j!^s)_{j\in \mathbb {N}}$, $s>0$, are violating (7.4).
- Consider the sequences $M^{q,n}:=(q^{j^n})_{j\in \mathbb {N}}$ with $q,n>1$. Then (7.4) is valid (with $A=B=1$ and *k* such that $k\ge 2^{1/(n-1)}$) as it is shown in [3, Example 6.6 (1)].
- Combining Lemma 7.1, Theorem 7.2 and Remark 4.6 yields the following: Let $M,L\in {\mathcal{L}\mathcal{C}}$ be given and assume that either *M* or *L* has $({\text {mg}})$ and that either *M* or *L* has (7.4). Then
  $$\begin{aligned} (4.3)\Longleftrightarrow F_M\preceq F_L\Longleftrightarrow F_L{\preceq _{\mathfrak {c}}}F_M\Longleftrightarrow (4.5), \end{aligned}$$
  i.e. in (4.7) the second implication can also be reversed.

#### Corollary 7.4

Let $M,L\in {\mathcal{L}\mathcal{C}}$ be given. Assume that both $F_M$ and $F_L$ satisfy the $\Delta _2$-condition. Then $F_{M\cdot L}$ and $F_{M\star L}$ satisfy the $\Delta _2$-condition, too.

#### Proof

By assumption *M* and *L* both have (7.4) and so it is immediate that $M\cdot L$ has this property as well.

Concerning the convolution note that we get $\omega _{M\star L}=\omega _M+\omega _L$ and so $\omega _{M\star L}$ has (7.3) because both $\omega _M$ and $\omega _L$ have this property. $\square$

Finally we apply Theorem 7.2 to $M=M^G$ and get the following.

#### Corollary 7.5

Let *G* be an N-function. Let $\omega _G$ be the function from (5.1), $M^G\in {\mathcal{L}\mathcal{C}}$ the associated sequence defined via (5.4) and $F_{M^G}$ the associated N-function. Then the following are equivalent:

(i) *G* satisfies the $\Delta _2$-condition.
(ii) The associated N-function $F_{M^G}$ satisfies the $\Delta _2$-condition.
(iii) $\varphi _{\omega _{M^G}}$ satisfies the $\Delta _2$-condition.
(iv) The function $\varphi ^G$ (see (5.13)) satisfies the $\Delta _2$-condition.
(v) $$\omega _G$$ (equivalently $$\omega _{M^G}$$) satisfies (7.2).
(vi) $\omega _G$ (equivalently $\omega _{M^G}$) satisfies (7.3).
(vii) $$M^G$$ satisfies (7.4).

#### Proof

Everything is immediate from Theorem 7.2 applied to $M^G$: $(i)\Leftrightarrow (ii)\Leftrightarrow (iii)\Leftrightarrow (iv)$ holds by Theorem 5.5 and since $\Delta _2$ is preserved under equivalence. Concerning (*v*) and (*vi*) note that condition (7.2) resp. (7.3) holds equivalently for $\omega _G$ and $\omega _{M^G}$ (by (5.5) and since these conditions are preserved under $\sim$). $\square$

### The $\nabla _2$-condition

Let *F* be an N-function and $F^c$ its complementary function. In [11, Thm. 4.2] and [17, Sect. 2.3, Thm. 3 (*a*)] it has been shown that $F^c$ satisfies the $\Delta _2$-condition if and only if *F* has

7.5 $$\begin{aligned} \exists \;\ell>1\;\exists \;t_0>0\;\forall \;t\ge t_0:\;\;\;2\ell F(t)\le F(\ell t), \end{aligned}$$

also known under the name $\nabla _2$ for *F* (see also [17, Sect. 2.3, Def. 2, p. 22]). A direct characterization for $\nabla ^2$ for *F* in terms of the left-derivative of *F* has been obtained in [17, Sect. 2.3, Thm. 3 (*b*)].

#### Remark 7.6

We summarize some properties for $\nabla _2$. For (*i*) and (*ii*) it is sufficient to require that $F,G:[0,+\infty )\rightarrow [0,+\infty )$ are non-decreasing, normalized and convex.

(i) $\nabla _2$ is preserved under relation $\sim _{\mathfrak {c}}$; this follows either from (*i*) in Remark 6.2 and since $\Delta _2$ is preserved under $\sim _{\mathfrak {c}}$, or it can be seen directly as follows: Assume that *F* has $\nabla _2$ and let *G* be another N-function such that $F{\sim _{\mathfrak {c}}}G$. So
  $$\begin{aligned} \exists \;k>1\;\exists \;t_0>0\;\forall \;t\ge t_0:\;\;\;G(tk^{-1})\le F(t)\le G(tk). \end{aligned}$$
  When iterating $\nabla _2$ we find (since $\ell >1$)
  $$\begin{aligned} \exists \;t_1>0\;\forall \;n\in \mathbb {N}_{>0}\;\forall \;t\ge t_1:\;\;\;F(t)\le \frac{1}{(2\ell )^n}F(\ell ^nt). \end{aligned}$$
  We choose $d\in \mathbb {N}_{>0}$ such that $\ell ^d\ge k^2$ and $n\in \mathbb {N}_{>0}$ such that $2^{n-1}\ge \ell ^d$. Then we estimate for all $t\ge \max \{t_0,t_1\}$ as follows:
  $$\begin{aligned} G(t)&\le F(kt)\le \frac{1}{(2\ell )^n}F(\ell ^n kt)\le \frac{1}{(2\ell )^n}F(\ell ^{n+d}k^{-1}t)\le \frac{1}{(2\ell )^n}G(\ell ^{n+d}t)\\&\le \frac{1}{2\ell ^{n+d}}G(\ell ^{n+d}t), \end{aligned}$$
  i.e. $\nabla _2$ for *G* holds with $\ell ^{n+d}$ and for all $t\ge t_2:=\max \{t_0,t_1\}$.
(ii) Similarly, we show that $\nabla _2$ is preserved under relation $\sim$: Assume that *F* has $\nabla _2$ and let *G* be another N-function such that $F\sim G$. So
  $$\begin{aligned} \exists \;k>1\;\exists \;t_0>0\;\forall \;t\ge t_0:\;\;\;k^{-1}G(t)\le F(t)\le kG(t), \end{aligned}$$
  and then we take $n\in \mathbb {N}_{>0}$ such that $k^2\le 2^{n-1}$. Iterating *n*-times property $\nabla _2$ gives for all *t* sufficiently large that
  $$\begin{aligned} \frac{1}{k}(2\ell )^nG(t)\le (2\ell )^nF(t)\le F(\ell ^nt)\le kG(\ell ^nt). \end{aligned}$$
  Since *G* is an N-function we get $k^2G(\ell ^nt)\le G(k^2\ell ^nt)$ (recall (3.2)) and $2k^2\ell ^n\le (2\ell )^n$ by the choice of *n*. Consequently, $2k^2\ell ^nG(t)\le G(k^2\ell ^nt)$ is verified for all sufficiently large *t*, i.e. $\nabla _2$ for *G* with $k^2\ell ^n$.
(iii) Let us prove that for any non-decreasing $F:[0,+\infty )\rightarrow [0,+\infty )$ with $\lim _{t\rightarrow +\infty }F(t)=+\infty$ we have that (7.5) is equivalent to
  7.6 $$\begin{aligned} \exists \;C\ge 1\;\exists \;\ell >1\;\forall \;t\ge 0:\;\;\;2\ell F(t)\le F(\ell t)+2\ell C. \end{aligned}$$
  (7.5) implies (7.6) with the same $\ell$, e.g. take $C:=F(t_0)$, because *F* is non-decreasing. For the converse first we iterate (7.6) and get $4\ell ^2F(t)\le 2\ell F(\ell t)+4\ell ^2C\le F(\ell ^2t)+2\ell C+4\ell ^2C$. Then, since $F(t)\rightarrow +\infty$ as $t\rightarrow +\infty$ we can find $t_0>0$ such that $F(\ell ^2t)\ge 2\ell C+4\ell ^2C$ for all $t\ge t_0$. Thus $4\ell ^2F(t)\le 2F(\ell ^2t)$ for all $t\ge t_0$ is verified, i.e. (7.5) with the choice $\ell ^2$.

In the weight sequence setting we are interested in having (7.5) for $F_M$ resp. for $\varphi _{\omega _M}$. Since $\nabla _2$ is preserved under equivalence, via Corollary 3.12 this condition transfers into

7.7 $$\begin{aligned} \exists \;\ell>1\;\exists \;s_0>1\;\forall \;s\ge s_0:\;\;\;2\ell \omega _M(s)\le \omega _M(s^{\ell }). \end{aligned}$$

The aim is to characterize now (7.7) in terms of *M*.

#### Theorem 7.7

Let $M\in {\mathcal{L}\mathcal{C}}$ be given. Then the following are equivalent:

(i) The associated N-function $F_M$ satisfies the $\nabla _2$-condition.
(ii) $\varphi _{\omega _M}$ satisfies the $\nabla _2$-condition.
(iii) $$\omega _M$$ satisfies (7.7).
(iv) $$\omega _M$$ satisfies
  7.8 $$\begin{aligned} \exists \;C\ge 1\;\exists \;\ell >1\;\forall \;s\ge 0:\;\;\;2\ell \omega _M(s)\le \omega _M(s^{\ell })+2\ell C. \end{aligned}$$
(v) The sequence *M* satisfies
  7.9 $$\begin{aligned} \exists \;A\ge 1\;\exists \;\ell >1\;\forall \;j\in \mathbb {N}:\;\;\;M_{2j}\le AM_j^{2\ell }. \end{aligned}$$

The proof shows that in (*iv*) and (*v*) we can take the same choice for $\ell$ and the correspondence between *C* and *A* is given by $A=e^{2\ell C}$. Consequently, if (7.8) holds for $\ell >1$ then also for all $\ell '\ge \ell$ (even with the same choice for *C*).

#### Proof

$(i)\Leftrightarrow (ii)$ follows from Corollary 3.12 and (*i*) in Remark 7.6. $(ii)\Leftrightarrow (iii)$ is clear and $(iii)\Leftrightarrow (iv)$ follows as in resp. by (*iii*) in Remark 7.6.

$(iv)\Rightarrow (v)$ By using (2.5) we get for all $j\in \mathbb {N}$:

$$\begin{aligned} M_{2j}&=\sup _{t\ge 0}\frac{t^{2j}}{\exp (\omega _M(t))}=\sup _{t\ge 0}\frac{t^{2j\ell }}{\exp (\omega _M(t^{\ell }))}\le e^{2\ell C}\sup _{t\ge 0}\frac{t^{2j\ell }}{\exp (2\ell \omega _M(t))} \\ {}&=e^{2\ell C}\left( \sup _{t\ge 0}\frac{t^j}{\exp (\omega _M(t))}\right) ^{2\ell }=e^{2\ell C}M_j^{2\ell }, \end{aligned}$$

so (7.9) is verified with $A:=e^{2\ell C}$ and the same $\ell$.

$(v)\Rightarrow (iv)$ We have $\frac{t^j}{M_j^{\ell }}\le \sqrt{A}\frac{t^j}{(M_{2j})^{1/2}}$ for all $j\in \mathbb {N}$ and $t\ge 0$. This yields by definition of associated weight functions

7.10 $$\begin{aligned} \forall \;t\ge 0:\;\;\;\omega _{M^{\ell }}(t)\le \omega _{\widetilde{M}^2}(t)+A_1, \end{aligned}$$

with $A_1:=\frac{\log (A)}{2}$, $M^{\ell }:=(M_j^{\ell })_{j\in \mathbb {N}}$ and the auxiliary sequence $\widetilde{M}^2:=(M_{2j}^{1/2})_{j\in \mathbb {N}}$, see [21, Sect.3], [20, (2.5), (2.6)] and also [4, Lemma 6.5]. When taking $c=2$ in [4, Lemma 6.5 (6.7)], then

7.11 $$\begin{aligned} \exists \;D\ge 1\;\forall \;t\ge 0:\;\;\;\omega _{\widetilde{M}^2}(t)\le 2^{-1}\omega _M(t)\le 2\omega _{\widetilde{M}^2}(t)+D. \end{aligned}$$

Using (2.4) and the first half of (7.11) we continue (7.10) and get

$$\begin{aligned} \forall \;t\ge 0:\;\;\;\ell \omega _M(t^{1/\ell })=\omega _{M^{\ell }}(t)\le \omega _{\widetilde{M}^2}(t)+A_1\le 2^{-1}\omega _M(t)+A_1, \end{aligned}$$

hence (7.8) is verified with the same $\ell$ and $C:=\frac{A_1}{\ell }=\frac{\log (A)}{2\ell }$. $\square$

#### Example 7.8

We provide some examples of sequences such that (7.9) is valid.

(i) Any $M\in {\mathcal{L}\mathcal{C}}$ with $({\text {mg}})$ does also have (7.9): By [18, Thm. 9.5.1] or [18, Thm. 9.5.3] applied to the matrix $\mathcal {M}=\{M\}$ (see also [13, Thm. 1]) we know that $({\text {mg}})$ is equivalent to
  7.12 $$\begin{aligned} \exists \;B\ge 1\;\forall \;j\in \mathbb {N}:\;\;\;M_{2j}\le B^jM_j^2. \end{aligned}$$
  Then note that $B^jM_j^2\le AM_j^{2\ell }\Leftrightarrow B\le A^{1/j}M_j^{2(\ell -1)/j}$ holds for all $j\in \mathbb {N}_{>0}$ if $A\ge 1$ is chosen sufficiently large because $\lim _{j\rightarrow +\infty }(M_j)^{1/j}=+\infty$ and $\ell >1$. Thus, in particular, the Gevrey sequences $G^s$, $s>0$, have (7.9).
(ii) However, the converse implication is not valid in general: Consider again the sequences $M^{q,n}:=(q^{j^n})_{j\in \mathbb {N}}$ with $q,n>1$. Condition (7.9) is valid because for given $n>1$ we choose $\ell \ge 2^{n-1}$ and get for all $j\in \mathbb {N}$ (and $q>1$) that
  $$\begin{aligned} q^{(2j)^n}=M^{q,n}_{2j}\le \big (M^{q,n}_j\big )^{2\ell }=\big (q^{j^n}\big )^{2\ell }\Leftrightarrow 1\le q^{2j^n(\ell -2^{n-1})}. \end{aligned}$$
  But (7.12) is violated: This requirement means $q^{(2j)^n}\le B^jq^{2j^n}$ and since $n>1$ this is impossible for any choice of *B* as $j\rightarrow +\infty$.

#### Corollary 7.9

Let $M,L\in {\mathcal{L}\mathcal{C}}$ be given and assume that both $F_M$ and $F_L$ satisfy the $\nabla _2$-condition. Then $F_{M\cdot L}$ and $F_{M\star L}$ have the $\nabla _2$-condition as well.

#### Proof

By assumption both sequences satisfy (7.9) and so it is immediate that $M\cdot L$ has this property, too.

Concerning the convolution we have $\omega _{M\star L}=\omega _M+\omega _L$ and so $\omega _{M\star L}$ has (7.8) because both $\omega _M$ and $\omega _L$ have this property. For this recall that if (7.8) holds for some $\ell >1$ then for all $\ell '\ge \ell$ as well. $\square$

Finally, let us apply Theorem 7.7 to $M=M^G$ from Sect. 5.

#### Corollary 7.10

Let *G* be an given N-function. Let $\omega _G$ be the associated weight function from (5.1), $M^G$ the associated weight sequence (see (5.4)) and finally $F_{M^G}$ the N-function associated with $M^G$. Then the following are equivalent:

(i) *G* satisfies the $\nabla _2$-condition (equivalently the complementary N-function $G^c$ satisfies the $\Delta _2$-condition).
(ii) $F_{M^G}$ satisfies the $\nabla _2$-condition (which is equivalent to the fact that the complementary N-function $F^c_{M^G}$ satisfies the $\Delta _2$-condition).
(iii) $\varphi _{\omega _{M^G}}$ satisfies the $\nabla _2$-condition (equivalently the complementary function $\varphi ^c_{\omega _{M^G}}$ satisfies the $\Delta _2$-condition).
(iv) The function $\varphi ^G$ (see (5.13)) satisfies the $\nabla _2$-condition.
(v) $$\omega _G$$ (equivalently $$\omega _{M^G}$$) satisfies (7.7).
(vi) $\omega _G$ (equivalently $\omega _{M^G}$) satisfies (7.8).
(vii) $$M^G$$ satisfies (7.9).

#### Proof

We apply Theorem 7.7 to $M^G$. Again $(i)\Leftrightarrow (ii)\Leftrightarrow (iii)\Leftrightarrow (iv)$ follows by Theorem 5.5 and the fact that both the $\Delta _2$ and the $\nabla _2$-condition are preserved under equivalence, recall (*i*) in Remark 7.6 for the latter one.

Concerning (*v*) and (*vi*) note that by (*ii*) in Remark 7.6 both (7.7) and (7.8) are preserved under relation $\sim$ which holds between $\omega _G$ and $\omega _{M^G}$ (recall (5.5)). $\square$

### The $\Delta ^2$-condition

According to [11, Chapter I, §6, 5] and [17, Def. 5, p. 40] we say that an N-function *F* satisfies the $\Delta ^2$-condition if

$$\begin{aligned} \exists \;k>1\;\exists \;t_0>0\;\forall \;t\ge t_0:\;\;\;F(t)^2\le F(kt). \end{aligned}$$

As stated on [11, p. 41], the $\Delta ^2$-condition is preserved under relation $\sim _{\mathfrak {c}}$ and $\Delta ^2$ holds if and only if $F^{\alpha }{\sim _{\mathfrak {c}}}F$ for some/any $\alpha >1$. In fact, this equivalence holds for any non-decreasing $F:[0,+\infty )\rightarrow [0,+\infty )$ with $\lim _{t\rightarrow +\infty }F(t)=+\infty$. By [11, Thm. 6.8] and [17, Sect. 2.5, Thm. 8] we know that *F* has $\Delta ^2$ if and only if the complementary function $F^c$ has

$$\begin{aligned} \exists \;k>1\;\exists \;t_0>0\;\forall \;t\ge t_0:\;\;\;F^c(t^2)\le ktF^c(t), \end{aligned}$$

also known as the $\nabla ^2$-condition, see e.g. [17, Def. 7, p. 41].

In view of Corollary 3.12 the associated N-function $F_M$ satisfies the $\Delta ^2$-condition if and only if

$$\begin{aligned} \exists \;k>1\;\exists \;t_0>0\;\forall \;t\ge t_0:\;\;\;(\omega _M(e^t))^2=\varphi _{\omega _M}(t)^2\le \varphi _{\omega _M}(kt)=\omega _M(e^{kt}), \end{aligned}$$

i.e.

7.13 $$\begin{aligned} \exists \;k>1\;\exists \;s_0>1\;\forall \;s\ge s_0:\;\;\;\omega _M(s)^2\le \omega _M(s^k). \end{aligned}$$

Note that (7.13) is not well-related to (2.5) and in general it seems to be difficult to obtain a characterization for $\Delta ^2$ in terms of *M* by using this formula.

However, we give the following characterization of $\Delta ^2$ in the weight sequence setting. First we have to prove a technical result:

#### Lemma 7.11

Let $M\in {\mathcal{L}\mathcal{C}}$ be given. Then the following are equivalent:

(i) The counting function $\Sigma _M$ satisfies
  7.14 $$\begin{aligned} \exists \;K>0\;\exists \;t_0>0\;\forall \;t\ge t_0:\;\;\;\Sigma _M(e^t)^2\le \Sigma _M(e^{tK}), \end{aligned}$$
  i.e. [11, (6.9)] for $p=\Sigma _M\circ \exp$ (this is the $\Delta ^2$-condition for $\Sigma _M\circ \exp$).
(ii) The sequence of quotients $\mu$ satisfies
  7.15 $$\begin{aligned} \exists \;A>0\;\exists \;j_0\in \mathbb {N}\;\forall \;j\ge j_0:\;\;\;\mu _{j^2}\le \mu _j^A. \end{aligned}$$

The proof shows the correspondence $A=K$.

#### Proof

$(i)\Rightarrow (ii)$ Write $s:=e^t$ and so $\Sigma _M(s)^2\le \Sigma _M(s^K)$ is satisfied for all $s\ge s_0:=e^{t_0}$. Let $j_0\in \mathbb {N}_{>0}$ be minimal such that $\mu _{j_0}\ge s_0$. Let $j\ge j_0$ be such that $\mu _j<\mu _{j+1}$ and take *s* with $\mu _j\le s<\mu _{j+1}$. Then $j^2=\Sigma _M(s)^2\le \Sigma _M(s^K)$ follows which implies $\mu _{j^2}\le s^K$. In particular, when taking $s:=\mu _j$ we have shown (7.15) with $A:=K$ and all $j\ge j_0$ such that $\mu _j<\mu _{j+1}$. If $j\ge j_0$ with $\mu _j=\dots =\mu _{j+\ell }<\mu _{j+\ell +1}$ for some $\ell \in \mathbb {N}_{>0}$, then following the previous step we get $\mu _{(j+\ell )^2}\le \mu _i^K$ for all $j\le i\le j+\ell$. Since $(j+\ell )^2\ge i^2$ for all such indices *i* and since by log-convexity $j\mapsto \mu _j$ is non-decreasing we are done for all $j\ge j_0$.

$(ii)\Rightarrow (i)$ Let $s\ge \mu _{j_0}$ and so $\mu _j\le s<\mu _{j+1}$ for some $j\ge j_0$. Then $\Sigma _M(s)=j$ and $s^A\ge \mu _j^A\ge \mu _{j^2}$ which implies $\Sigma _M(s^A)\ge j^2=\Sigma _M(s)^2$. Thus (7.14) is shown with $K:=A$ and $t_0:=\log (\mu _{j_0})$. $\square$

Using this we get the following characterization.

#### Theorem 7.12

Let $M\in {\mathcal{L}\mathcal{C}}$ be given. Then the following are equivalent:

(i) The counting function $\Sigma _M$ satisfies (7.14), i.e. so $\Sigma _M\circ \exp$ satisfies the $\Delta ^2$-condition.
(ii) The sequence of quotients $\mu$ satisfies (7.15).
(iii) The associated N-function $F_M$ (or equivalently $\varphi _{\omega _M}$) satisfies the $\Delta ^2$-condition.

#### Proof

$(i)\Leftrightarrow (ii)$ is verified in Lemma 7.11.

$(i)\Rightarrow (iii)$ In view of (3.19) the function $f_M$ appearing in the representation (3.1) of $F_M$ enjoys the $\Delta ^2$-condition (with the same *K* and for all $t\ge t_1$, $t_1$ possibly strictly larger than $t_0$ appearing in (7.14)). Thus we can apply [11, Lemma 6.1, Thm. 6.4] in order to conclude. There the estimate in (7.14) is assumed to be strict; however the proof only requires $\le$.

$(iii)\Rightarrow (i)$ We repeat the arguments from [17, Sect. 2.5, Thm. 8. $(i)\Rightarrow (ii)$] (for the representation involving the left-continuous density for $F_M$). First, since $F_M$ satisfies the $\Delta ^2$-condition it also has the $\Delta _3$-condition, see Sect. 7.4. Consequently, by taking into account Lemma 7.17 and Theorem 7.18 we get that $\Sigma _M\circ \exp$ resp. equivalently $f_M$ satisfies the $\Delta _3$-condition, too. Summarizing, we have

7.16 $$\begin{aligned} \exists \;k>1\;\exists \;t_0>0\;\forall \;t\ge t_0:\;\;\;tf_M(t)\le f_M(kt),\hspace{30pt}F_M(t)^2\le F_M(kt).\nonumber \\ \end{aligned}$$

Using the second part of this and since $F_M(2x)=\int _0^{2x}f_M(s)ds\ge \int _x^{2x}f_M(s)ds\ge f_M(x)x$ for all $x\ge 0$ we estimate as follows for all $x>0$ with $x\ge t_0$:

$$\begin{aligned} f_M(x)^2\le \frac{1}{x^2}F_M(2x)^2\le \frac{1}{x^2}F_M(2kx)=\frac{1}{x^2}\int _0^{2kx}f_M(s)ds\le \frac{1}{x^2}f_M(2kx)2kx. \end{aligned}$$

If $x\ge \max \{1,t_0\}$, then this estimate and the first part in (7.16) applied to $t:=2kx(>t_0)$ give

$$\begin{aligned} f_M(x)^2\le 2kxf_M(2kx)\le f_M(2k^2x), \end{aligned}$$

i.e. the $\Delta ^2$-condition for $f_M$ is verified with $t_1:=\max \{1,t_0\}$ and $k_1:=2k^2$. By (3.19) this is equivalent to the fact that $\Sigma _M\circ \exp$ satisfies the $\Delta ^2$-condition; i.e. (7.14) for $\Sigma _M$. $\square$

#### Corollary 7.13

Let *G* be an N-function and let $M^G$ be the associated weight sequence (see (5.4)). Then the following are equivalent:

(i) The sequence of quotients $\mu ^G$ satisfies (7.15).
(ii) *G* satisfies the $\Delta ^2$-condition.
(iii) The associated N-function $F_{M^G}$ satisfies the $\Delta ^2$-condition.
(iv) The function $\varphi ^G$ (see (5.13)) satisfies the $\Delta ^2$-condition.

Recall that in order to verify (7.15) for $\mu ^G$ for abstractly given N-functions *G* the formula (5.10) can be used.

#### Proof

First, Theorem 7.12 applied to $M^G$ yields $(i)\Leftrightarrow (iii)$. By Theorem 5.5 we have that $F_{M^G}$, *G* and $\varphi ^G$ are equivalent and since $\Delta ^2$ is preserved under equivalence we are done. $\square$

#### Remark 7.14

We gather some observations:

(i) (7.15) means that the sequence of quotients has to increase “relatively slowly” and w.l.o.g. we can assume $A\in \mathbb {N}_{\ge 2}$ in this condition (since $\mu _j\ge 1$ for all *j*).
(ii) (7.15) is preserved under relation $\cong$: Let $M,L\in {\mathcal{L}\mathcal{C}}$ such that $M\cong L$ and assume that *M* has (7.15). Then
  $$\begin{aligned} \exists \;A>0\;\exists \;B\ge 1\;\exists \;j_0\in \mathbb {N}\;\forall \;j\ge j_0:\;\;\;\frac{1}{B}\lambda _{j^2}\le \mu _{j^2}\le \mu _j^A\le B\lambda _j^A \end{aligned}$$
  follows and since $\lim _{j\rightarrow +\infty }\lambda _j=+\infty$ we get $B^2\lambda _j^A\le \lambda _j^{A'}$ for any $A'>A$ and for all $j\ge j_{A',B}$ sufficiently large. Thus *L* satisfies (7.15) when choosing $A'>A$ and restricting to $j\ge \max \{j_0,j_{A',B}\}$.
(iii) In [11, (6.10), p. 43] another sufficiency criterion is given. Suppose that an N-function *F* has
  $$\begin{aligned} \exists \;\alpha>0\;\exists \;t_0>0:\;\;\;t\mapsto \frac{\log (F(t))}{t^{\alpha }}\;\;\;\text {is not decreasing on}\;[t_0,+\infty ), \end{aligned}$$
  then *F* satisfies the $\Delta ^2$-condition. One verifies that this condition yields $F^2{\sim _{\mathfrak {c}}}F$ and hence this implication holds for any non-decreasing $F:[0,+\infty )\rightarrow [0,+\infty )$ with $\lim _{t\rightarrow +\infty }F(t)=+\infty$. In the weight sequence setting this expression amounts to the study of $\frac{\log (\varphi _{\omega _M}(t))}{t^{\alpha }}=\frac{\log (\omega _M(e^t))}{t^{\alpha }}=\frac{\log (\omega _M(s))}{\log (s)^{\alpha }}$ for all $s\ge s_0=e^{t_0}$. If there exists $\alpha >0$ such that $s\mapsto \frac{\log (\omega _M(s))}{\log (s)^{\alpha }}$ is non-decreasing for all large *s*, then $\varphi _{\omega _M}$ satisfies the $\Delta ^2$-condition and so $F_M$ as well.

#### Example 7.15

We comment on some examples.

- All Gevrey-sequences $G^s$, $s>0$, satisfy (7.15): This condition amounts to $(j^2)^s\le (j^s)^A$ and so the choices $A:=2$ and $j_0:=0$ are sufficient (for any $s>0$).
- If $M,L\in {\mathcal{L}\mathcal{C}}$ both have (7.15), then also the product sequence $M\cdot L$: The corresponding sequence of quotients is given by the product $\mu \cdot \lambda$ and so (7.15) follows immediately. The same implication is not clear for the convolution product $M\star L$.
- The sequence $M^{q,2}$ does not satisfy this requirement because in this case the corresponding sequence of quotients is given by $(q^{2j-1})_j$. Then (7.15) transfers into $q^{2j^2-1}\le q^{(2j-1)A}$ but which is impossible for any choice $A\ge 1$ if $j\rightarrow +\infty$. Alternatively, one can also show that in this case (7.13) is violated: We have, see the proof of Corollary 7.20 for more details and citations, that $\omega _{M^{q,2}}\sim \omega _2$ for all $q>1$, i.e. $\omega _{M^{q,2}}(t)=O(\omega _2(t))$ and $\omega _2(t)=O(\omega _{M^{q,2}}(t))$ as $t\rightarrow +\infty$ for each $q>1$ with $\omega _2(t):=\max \{0,\log (t)^2\}$. Fix $q>1$ and so (7.13) gives for some $C\ge 1$ and all $s\ge 1$
  $$\begin{aligned} -1+C^{-1}\log (s)^4\le \omega _{M^{q,2}}(s)^2\le \omega _{M^{q,2}}(s^k)\le C\log (s^k)^2+C=Ck^2\log (s)^2+C, \end{aligned}$$
  yielding a contradiction as $s\rightarrow +\infty$.

Summarizing, by Examples 7.3, 7.8 and 7.15 we get the following consequences which should be compared with the first diagram on [17, p. 43]:

7.17 $$\begin{aligned} \Delta _2\wedge \nabla _2,\Delta _2,\nabla _2\nRightarrow \Delta ^2,\hspace{25pt}\Delta ^2\nRightarrow \Delta _2,\hspace{25pt}\nabla _2\nRightarrow \Delta _2. \end{aligned}$$

Let us study how condition (7.15) is related to moderate growth.

#### Lemma 7.16

Let $M\in {\mathcal{L}\mathcal{C}}$ be given such that $({\text {mg}})$ holds and

7.18 $$\begin{aligned} \liminf _{n\rightarrow +\infty }(\mu _{2^n})^{1/(n+2)}>1. \end{aligned}$$

Then *M* satisfies (7.15) and hence the associated N-function $F_M$ (or equivalently $\varphi _{\omega _M}$) satisfies the $\Delta ^2$-condition.

#### Proof

First, see e.g. [16, Lemma 2.2] and the citations there, $({\text {mg}})$ is equivalent to $\sup _{j\in \mathbb {N}}\frac{\mu _{2j}}{\mu _j}<+\infty$. Consequently, we find some $A>1$ such that $\mu _{2^kj}\le A^k\mu _j$ for each $k,j\in \mathbb {N}$.

Take now $j\in \mathbb {N}_{>0}$ (the case $j=0$ is trivial), then $2^n\le j<2^{n+1}$ for some $n\in \mathbb {N}$ and $2^{2n}\le j^2<2^{2n+2}$, so

$$\begin{aligned} \mu _{j^2}\le \mu _{2^{2n+2}}=\mu _{2^{n+2}2^n}\le A^{n+2}\mu _{2^n}. \end{aligned}$$

By (7.18) we find $n_0\in \mathbb {N}$ and $\delta >1$ such that $(\mu _{2^n})^{1/(n+2)}\ge \delta$ for all $n\ge n_0$. Set $B:=\frac{\log (A)}{\log (\delta )}+1>1$, so $\delta =A^{1/(B-1)}$ and hence $A^{n+2}\le \mu _{2^n}^{B-1}$ for all $n\ge n_0$. This implies $\mu _{j^2}\le A^{n+2}\mu _{2^n}\le \mu _{2^n}^B\le \mu _j^B$ and so (7.15) is verified (for $j_0:=2^{n_0}$ and choosing *B* as before). $\square$

We finish by commenting on requirement (7.18):

- (7.18) is a mild extra growth assumption: It follows when $\liminf _{j\rightarrow \infty }\frac{\mu _j}{j}>0$ because then $\mu _j\ge j\epsilon$ for some $\epsilon >0$ and all $j\in \mathbb {N}$. Thus $(\mu _{2^n})^{1/(n+2)}\ge 2^{n/(n+2)}\epsilon ^{1/(n+2)}$ for all $n\in \mathbb {N}$ and so $(\mu _{2^n})^{1/(n+2)}>1$ for all *n* sufficiently large (depending on $\epsilon$).
- On the other hand note that $\lim _{n\rightarrow +\infty }(2^{ns})^{1/(n+2)}=2^s>1$ for any $s>0$ and hence each $G^s$ satisfies (7.18). However, $\lim _{j\rightarrow +\infty }\frac{j^s}{j}=0$ holds for all $0<s<1$.

### The $\Delta _3$-condition

An N-function *F* satisfies the $\Delta _3$-condition, see [11, Chapter I, §6, (6.1)] and [17, Sect. 2.5, Def. 1, p. 37], if

7.19 $$\begin{aligned} \exists \;k>1\;\exists \;t_0>0\;\forall \;t\ge t_0:\;\;\;tF(t)\le F(kt). \end{aligned}$$

Since $F(t)\ge t$ for all large *t* (recall the second part in (3.3)) we immediately have that $\Delta ^2$ implies $\Delta _3$; however the converse is not true in general, see [11, p. 41] and [17, p. 40]. It is also known that $\Delta _3$ for *F* implies $\Delta _2$ for $F^c$, i.e. *F* has $\nabla _2$, see [11, Thm. 6.5]. Moreover, by [17, Sect. 2.5, Thm. 3] we have that *F* has $\Delta _3$ if and only if $F^c$ satisfies the $\nabla _3$-condition, see again [17, Sect. 2.5, Def. 1, p. 37].

$\Delta _3$ is preserved under equivalence and since $tF(t)\ge F(t)$ for all $t\ge 1$ we get that

- *F* has $\Delta _3$ if and only if
- *F* and $t\mapsto tF(t)$ are equivalent,

see [11, Chapter I, §6, 1, p. 35]. Moreover, we have the following reformulation for $\Delta _3$:

#### Lemma 7.17

Let *F* be an N-function. Then the following are equivalent:

(i) *F* satisfies the $\Delta _3$-condition.
(ii) We have $\widetilde{F}{\sim _{\mathfrak {c}}}F$ with
  7.20 $$\begin{aligned} \widetilde{F}(t):=\int _0^{|t|}F(s)ds,\;\;\;t\in \mathbb {R}. \end{aligned}$$
(iii) The function *f* from the representation (3.1) satisfies the $\Delta _3$-condition.
(iv) We have that $F{\sim _{\mathfrak {c}}}f$.

Consequently, if any of these equivalent conditions holds, then $\widetilde{F}$ has $\Delta _3$, too.

#### Proof

$(i)\Rightarrow (ii)$ This is contained in the proof of [11, Thm. 6.1]. First, for any N-function *F* we get for all $t\ge 1$ that

$$\begin{aligned} \widetilde{F}(2t)=\int _0^{2t}F(s)ds\ge \int _t^{2t}F(s)ds\ge F(t)t\ge F(t). \end{aligned}$$

In fact this holds for any non-decreasing and non-negative *F*. On the other hand, by using $\Delta _3$ for some $k>1$ and all *t* sufficiently large one has

$$\begin{aligned} \widetilde{F}(t)=\int _0^tF(s)ds\le F(t)t\le F(kt). \end{aligned}$$

$(ii)\Rightarrow (i)$ By the equivalence we get $\widetilde{F}(t)\le F(kt)$ for some $k>1$ and all *t* sufficiently large. Thus for all such large *t* we estimate by

$$\begin{aligned} F(k2t)\ge \widetilde{F}(2t)=\int _0^{2t}F(s)ds=\int _0^tF(s)ds+\int _t^{2t}F(s)ds\ge \widetilde{F}(t)+F(t)t\ge F(t)t, \end{aligned}$$

i.e. $$\Delta _3$$ with $$k':=2k$$.

$(i)\Rightarrow (iii)$ This is shown in the proof of [17, Sect. 2.5, Thm. 3. $(i)\Rightarrow (ii)$] (for the representation involving the left-continuous density for *F*); we repeat the details: First, we estimate by

$$\begin{aligned} xf(x)\le & {} \int _x^{2x}f(s)ds\le \int _0^{2x}f(s)ds=F(2x)\le \frac{1}{2x}F(2kx)\\= & {} \frac{1}{2x}\int _0^{2kx}f(s)ds\le \frac{1}{2x}2kxf(2kx), \end{aligned}$$

thus $xf(x)\le 2kf(2kx)$ for all $x\ge t_0/2$, with $k>1$, $t_0>0$ from (7.19). We use this estimate and apply it also to $y:=2kx(>t_0)$ in order to get

$$\begin{aligned} xf(x)\le 2kf(2kx)\le xf(2kx)\le f(4k^2x) \end{aligned}$$

for all $x\ge t_1:=\max \{t_0,2k\}$. Thus $\Delta _3$ for *f* is verified with $t_1$ and $k_1:=4k^2$.

$(iii)\Rightarrow (i),(iv)$ We replace in $(i)\Rightarrow (ii)$ the function *F* by *f* and $\widetilde{F}$ by *F* (i.e. (7.20) turns into (3.1)).

$(iv)\Rightarrow (iii)$ This follows by replacing in $(ii)\Rightarrow (i)$ the function *F* by *f* and $\widetilde{F}$ by *F*. $\square$

We apply this characterization to the weight sequence setting.

#### Theorem 7.18

Let $M\in {\mathcal{L}\mathcal{C}}$ be given. Then the following are equivalent:

(i) The associated N-function $F_M$ (equivalently $\varphi _{\omega _M}$) satisfies the $\Delta _3$-condition.
(ii) The function $\Sigma _M\circ \exp$ (equivalently $f_M$) satisfies the $\Delta _3$-condition.
(iii) $\widetilde{F}_M{\sim _{\mathfrak {c}}}F_M$ is valid (with $\widetilde{F}_M$ given by (7.20)).
(iv) $\widetilde{\varphi }_{\omega _M}{\sim _{\mathfrak {c}}}\varphi _{\omega _M}$ holds with
  $$\begin{aligned} \widetilde{\varphi }_{\omega _M}(t):=\int _0^{|t|}\varphi _{\omega _M}(s)ds,\;\;\;t\in \mathbb {R}. \end{aligned}$$
(v) We have that
  7.21 $$\begin{aligned} \exists \;k>1\;\exists \;s_0>1\;\forall \;s\ge s_0:\;\;\;\omega _M(s)\le \widetilde{\omega }_M(s^2)\le \omega _M(s^k), \end{aligned}$$
  with
  $$\begin{aligned} \widetilde{\omega }_M(t):=\widetilde{\varphi }_{\omega _M}(\log (t)),\;\;\;t\ge 1,\hspace{15pt}\widetilde{\omega }_M(t):=0,\;\;\;0\le t<1. \end{aligned}$$

The function $\widetilde{\omega }_M$ admits the representation

7.22 $$\begin{aligned} \widetilde{\omega }_M(s)=\int _0^{|s|}\frac{\omega _M(u)}{u}du=\int _1^{|s|}\frac{\omega _M(u)}{u}du,\;\;\;s\in \mathbb {R}. \end{aligned}$$

#### Proof

$(i)\Leftrightarrow (ii)\Leftrightarrow (iii)$ follows by Lemma 7.17 applied to $F_M$, by (3.19) and the fact that $\Delta _3$ is preserved under equivalence, see Corollary 3.12.

$(iii)\Rightarrow (iv)$ The estimate $\widetilde{\varphi }_{\omega _M}(2t)\ge \varphi _{\omega _M}(t)$ for $t\ge 1$ holds as in $(i)\Rightarrow (ii)$ in Lemma 7.17 and for the converse we estimate as follows for *t* sufficiently large:

$$\begin{aligned} \widetilde{\varphi }_{\omega _M}(t)&=\int _0^{t}\varphi _{\omega _M}(s)ds\le \int _0^t(F_M(s)+C)ds=\widetilde{F}_M(t)+Ct\le F_M(kt)+Ct \\ {}&\le \varphi _{\omega _M}(kt)+Ct+D\le 2\varphi _{\omega _M}(kt)\le \varphi _{\omega _M}(2kt). \end{aligned}$$

The first estimate follows for some $C\ge 1$ (and all $s\ge 0$) by (3.18), the second one since $\widetilde{F}_M{\sim _{\mathfrak {c}}}F_M$ by assumption, the third one again by (3.18), the fourth since $\lim _{t\rightarrow +\infty }\frac{\varphi _{\omega _M}(t)}{t}=+\infty$, and finally the last one by convexity and normalization for $\varphi _{\omega _M}$ (see (3.2)).

$(iv)\Rightarrow (iii)$ $\widetilde{F}_M(2t)\ge F_M(t)$ for all $t\ge 1$ is shown in $(i)\Rightarrow (ii)$ in Lemma 7.17. Conversely, by assumption $\widetilde{\varphi }_{\omega _M}(t)\le \varphi _{\omega _M}(kt)$ for some $k>1$ and all $t(\ge 1)$ large. Thus for all *t* sufficiently large:

$$\begin{aligned} \widetilde{F}_M(t)&=\int _0^{t}F_M(s)ds\le \int _0^t(\varphi _{\omega _M}(s)+D)ds=\widetilde{\varphi }_{\omega _M}(t)+Dt\le \varphi _{\omega _M}(kt)+Dt \\ {}&\le F_M(kt)+Dt+C\le 2F_M(kt)\le F_M(2kt). \end{aligned}$$

The first estimate follows by (3.18) (for all $s\ge 0$), the second one by assumption, the third one again by (3.18), for the fourth estimate we have used the second part in (3.3) and finally the last one holds by (3.2).

$(iv)\Leftrightarrow (v)$ First, for all $t\ge 0$ we have

$$\begin{aligned} \widetilde{\omega }_M(e^t)=\widetilde{\varphi }_{\omega _M}(t)=\int _0^t\omega _M(e^s)ds=\int _1^{e^t}\frac{\omega _M(u)}{u}du=\int _0^{e^t}\frac{\omega _M(u)}{u}du, \end{aligned}$$

because $\omega _M(t)=0$ for $0\le t\le 1$. Thus $\widetilde{\omega }_M(t)=\int _0^t\frac{\omega _M(u)}{u}du$ for all $t\ge 1$ and in fact even for all $t\ge 0$ (since $\widetilde{\omega }_M(t):=0$ for $0\le t\le 1$). Thus (7.22) is verified.

Moreover, $\widetilde{\varphi }_{\omega _M}{\sim _{\mathfrak {c}}}\varphi _{\omega _M}$ holds if and only if

7.23 $$\begin{aligned} \exists \;k>1\;\exists \;t_0>0\;\forall \;t\ge t_0:\;\;\;\varphi _{\omega _M}(t)\le \widetilde{\varphi }_{\omega _M}(2t)\le \varphi _{\omega _M}(kt), \end{aligned}$$

since the first estimate holds for all $t\ge 1$ as mentioned in $(ii)\Rightarrow (iii)$. (7.23) is obviously equivalent to (7.21). $\square$

Using this characterization we give two applications.

#### Corollary 7.19

Let $M,L\in {\mathcal{L}\mathcal{C}}$ be given. If both $F_M$ and $F_L$ have the $\Delta _3$-condition, then $F_{M\star L}$, too.

#### Proof

Recall that $M\star L\in {\mathcal{L}\mathcal{C}}$ and $\omega _{M\star L}=\omega _M+\omega _L$. Then (7.22) implies $\widetilde{\omega }_{M\star L}=\widetilde{\omega }_M+\widetilde{\omega }_L$ and by assumption we have (7.21) for both $\omega _M$ and $\omega _L$ and so for $\omega _{M\star L}$ as well. Theorem 7.18 yields that $F_{M\star L}$ satisfies the $\Delta _3$-condition, too. $\square$

#### Corollary 7.20

There exist N-functions having $\Delta _2$ and $\nabla _2$ but not $\Delta _3$.

This statement should be compared with the first diagram on [17, p. 43].

#### Proof

Let us consider the sequence(s) $M^{q,n}$ with $q,n>1$. As seen in Examples 7.3, 7.8 each sequence yields an associated N-function $F_{M^{q,n}}$ having both $\Delta _2$ and $\nabla _2$.

We prove now that (7.21) is violated. For this first recall results from [16, Sect. 5.5] and [18, Sect. 3.10]: Let $n>1$ be arbitrary but from now on fixed, then each $M^{q,n}$ is an element of the weight matrix (i.e. the one-parameter family of weight sequences) associated with the weight $\omega _s(t):=\max \{0,\log (t)^s\}$, $s>1$, such that $\frac{1}{s}+\frac{1}{n}=1$. Therefore, $\omega _{M^{q,n}}\sim \omega _s$ for all $q>1$, i.e. $\omega _{M^{q,n}}(t)=O(\omega _s(t))$ and $\omega _s(t)=O(\omega _{M^{q,n}}(t))$ as $t\rightarrow +\infty$ for each $q>1$, see [18, Thm. 4.0.3] and [15, Lemma 5.7].

For all $x\ge 1$ we have

$$\begin{aligned} \int _0^x\frac{\omega _s(t)}{t}dt=\int _1^x\frac{\log (t)^s}{t}dt=\left[ \frac{\log (t)^{s+1}}{s+1}\right] _{t=1}^{t=x}=\frac{\log (x)^{s+1}}{s+1}, \end{aligned}$$

and so by the above (recall also (7.22))

$$\begin{aligned} \forall \;q>1\;\exists \;D\ge & {} 1\;\forall \;x\ge 1:\;\;\; D^{-1}\frac{\log (x)^{s+1}}{s+1}-\log (x)\\\le & {} \widetilde{\omega }_{M^{q,n}}(x)\le D\frac{\log (x)^{s+1}}{s+1}+D\log (x). \end{aligned}$$

Fix now $q>1$ and then, when (7.21) is valid, we obtain

$$\begin{aligned}&D^{-1}\frac{\log (x^2)^{s+1}}{s+1}-\log (x^2)\le \widetilde{\omega }_{M^{q,n}}(x^2)\le \omega _{M^{q,n}}(x^k)\le D\log (x^k)^s+D \\ {}&\Longrightarrow 2^{s+1}\log (x)^{s+1}\le D^2(s+1)k^s\log (x)^s+D^2(s+1)+2(s+1)D\log (x). \end{aligned}$$

But this is impossible as $x\rightarrow +\infty$ for any choices of *D* and *k*. $\square$

By applying Theorem 7.18 to the associated sequence $M^G$, Proposition 5.4, Theorem 5.5 and recalling that $\Delta _3$ is preserved under equivalence we obtain:

#### Corollary 7.21

Let *G* be an N-function and let $M^G$ be the associated sequence (see (5.4)). Then the following are equivalent:

(i) *G* satisfies the $\Delta _3$-condition.
(ii) The associated N-function $F_{M^G}$ satisfies the $\Delta _3$-condition.
(iii) $\varphi ^G$ (see (5.13)) satisfies the $\Delta _3$-condition.
(iv) The function $\Sigma _{M^G}\circ \exp$ (equivalently $f_{M^G}$) satisfies the $\Delta _3$-condition.
(v) The function $\Sigma ^G\circ \exp$ (see (5.11)) satisfies the $\Delta _3$-condition.
(vi) Assertions (*iv*) resp. (*v*) listed in Theorem 7.18 are valid for the associated sequence $M^G$.

We close with the following observation concerning assertion (*ii*) in Theorem 7.18.

#### Lemma 7.22

Let $M\in {\mathcal{L}\mathcal{C}}$ be given. Consider the following conditions:

(i) The sequence of quotients $\mu$ satisfies
  $$\begin{aligned} \exists \;k>1\;\exists \;j_0\in \mathbb {N}\;\forall \;j\ge j_0:\;\;\;\mu _j^k\ge \mu _{\lceil j\log (\mu _{j+1})\rceil }. \end{aligned}$$
(ii) The function $\Sigma _M\circ \exp$ (equivalently $f_M$) satisfies the $\Delta _3$-condition.
(iii) The sequence of quotients $\mu$ satisfies
  $$\begin{aligned} \exists \;k>1\;\exists \;j_0\in \mathbb {N}\;\forall \;j\ge j_0:\;\;\;\mu _j^k\ge \mu _{\lceil j\log (\mu _{j})\rceil }. \end{aligned}$$

Then $$(i)\Rightarrow (ii)\Rightarrow (iii)$$ holds.

#### Proof

$(i)\Rightarrow (ii)$ Let $t\ge 0$ be such that $\mu _j\le t<\mu _{j+1}$ for some $j\ge j_0$. Then $\Sigma _M(t)=j$ and $t^k\ge \mu _j^k\ge \mu _{\lceil j\log (\mu _{j+1})\rceil }$, hence $\Sigma _M(t^k)\ge \lceil j\log (\mu _{j+1})\rceil \ge j\log (\mu _{j+1})=\Sigma _M(t)\log (\mu _{j+1})\ge \Sigma _M(t)\log (t)$. Hence the $\Delta _3$-condition is verified with the same *k* and $t_0:=\mu _{j_0}$.

$(ii)\Rightarrow (iii)$ Let $j\in \mathbb {N}$ such that $\mu _{j+1}>\mu _j\ge t_0$ with $t_0$ the value appearing in the $\Delta _3$-condition. Then this property evaluated at $t=\mu _j$ yields $\log (\mu _j)j=\log (\mu _j)\Sigma _M(\mu _j)\le \Sigma _M(\mu _j^k)$ which gives the desired estimate with the same *k* since $\Sigma _M(\mu _j^k)\in \mathbb {N}$. If $\mu _j\ge t_0$ such that $\mu _j=\dots =\mu _{j+d}<\mu _{j+d+1}$, then $t=\mu _j$ yields $\log (\mu _{j+i})(j+i)\le \log (\mu _{j+i})(j+d)=\log (t)\Sigma _M(t)\le \Sigma _M(t^k)$ for all $0\le i\le d$ and so, since $\mu _j^k=\dots =\mu _{j+d}^k$ and $\mu$ is non-decreasing, we get $\mu _{j+i}^k\ge \mu _{\lceil \log (\mu _{j+i})(j+i)\rceil }$ for all $0\le i\le d$. Thus we are done by taking the same *k* and $j_0\in \mathbb {N}$ minimal such that $\mu _{j_0}\ge t_0$. $\square$

### The $\Delta '$-condition

According to [11, Chapter I, §5] and [17, Sect. 2.3, Def. 7, p. 28] we say that an N-function *F* satisfies the $\Delta '$-condition if

$$\begin{aligned} \exists \;k>0\;\exists \;u_0>0\;\forall \;t,s\ge u_0:\;\;\;F(ts)\le kF(t)F(s). \end{aligned}$$

In the weight sequence setting in view of Corollary 3.12 and since $\Delta '$ is preserved under equivalence, see [11, Chapter I, §5, p. 30], this condition means that

7.24 $$\begin{aligned} \exists \;k>0\;\exists \;u_0>0\;\forall \;t,s\ge u_0:\;\;\;\omega _M(t^{\log (s)})\le k\omega _M(s)\omega _M(t). \end{aligned}$$

By [11, Lemma 5.1] and [17, Sect. 2.3, Lemma 8] we know that $\Delta '$ implies $\Delta _2$ and on [11, p. 30–31] (see also [17, p. 29]) it is shown that in general this implication is strict. Moreover, by [11, Thm. 6.6] it follows that if *F* satisfies $\Delta ^2$, then $F^c$ has $\Delta '$ and by [17, Sect. 2.3, Thm. 11] we know that *F* has $\Delta '$ if and only if $F^c$ satisfies the so-called $\nabla '$-condition; see [17, Sect. 2.3, Def. 7, p. 28 (11)]. [17, Sect. 2.3, Prop. 12] tells us that an N-function $F\in \Delta '\cap \nabla '$ if and only if *F* is equivalent to $t\mapsto |t|^s$ for some $s>1$; i.e. this corresponds to the weight $\omega _s(t):=\max \{0,\log (t)^s\}$ and hence to the sequences $M^{q,n}$ such that $\frac{1}{s}+\frac{1}{n}=1$, see the proof of Corollary 7.20.

A direct check of (7.24) seems to be quite technical resp. hardly possible since due to the multiplicative nature this estimate is not well-related w.r.t. formula (2.5). The same comment applies to $\nabla '$ and to the counting function $\Sigma _M$.

In [11, Thm. 5.1] a sufficiency criterion for $\Delta '$ is shown:

#### Theorem 7.23

Let $F(t)=\int _0^{|t|}f(s)ds$ be a given N-function (recall the representation (3.1)). Then *F* satisfies the $\Delta '$-condition provided that *f* has the following growth property which we abbreviate by $(\Delta '_f)$ from now on:

There exists some $t_0>1$ such that for every fixed $t\ge t_0$ the function $h_f$ given by $h_f(s):=\frac{f(st)}{f(s)}$ is not increasing on $[t_0,+\infty )$.

In the weight sequence setting this result takes the following form:

#### Corollary 7.24

Let $M\in {\mathcal{L}\mathcal{C}}$ be given. If $\Sigma _M\circ \exp$ satisfies $(\Delta '_{\Sigma _M\circ \exp })$ then the associated N-function $F_M$ (resp. equivalently $\varphi _{\omega _M}$) satisfies the $\Delta '$-condition.

#### Proof

In view of (3.19), we get $(\Delta '_{\Sigma _M\circ \exp })$ if and only if $(\Delta '_{f_M})$ when enlarging $t_0$ sufficiently if necessary. Then Theorem 7.23 applied to $f=f_M$ and $F=F_M$ yields the conclusion. $\square$

However, we show now that in general $(\Delta '_{\Sigma _M\circ \exp })$ fails in the weight sequence setting.

#### Proposition 7.25

Let $M\in {\mathcal{L}\mathcal{C}}$ be given such that $1\le \mu _1<\mu _2<\dots$, i.e. the sequence of quotients is strictly increasing (see Remark 2.2). Then $(\Delta '_{\Sigma _M\circ \exp })$ (resp. equivalently $(\Delta '_{f_M})$) is violated.

#### Proof

First, with $u:=e^s$ we get $\frac{\Sigma _M(e^{ts})}{\Sigma _M(e^s)}=\frac{\Sigma _M(u^t)}{\Sigma _M(u)}$ and hence $(\Delta '_{\Sigma _M\circ \exp })$ precisely means:

$$\begin{aligned} \exists \;t_0>1\;\forall \;t\ge t_0:\;\;\;u\mapsto \frac{\Sigma _M(u^t)}{\Sigma _M(u)}\;\text {is not increasing on}\;\;\;[e^{t_0},+\infty )=:[u_0,+\infty ). \end{aligned}$$

Let now $t\ge t_0>1$ be arbitrary but fixed. Then $\mu _{j_0}\le u_0<\mu _{j_0+1}$ and $\mu _k\le u_0^t<\mu _{k+1}$ for some (large) $j_0,k\in \mathbb {N}_{>0}$. Note that *k* is depending on *t* and $\frac{\Sigma _M(u_0^t)}{\Sigma _M(u_0)}=\frac{k}{j_0}$. Since $u_0^t\ge u_0$ we clearly have $k\ge j_0$.

We show that assuming $(\Delta '_{\Sigma _M\circ \exp })$ yields a contradiction. Let $u\in [u_0,+\infty )$ increase and we split the argument in several steps:

- If $u\ge u_0$ is given with $\mu _{j_0}<u<\mu _{j_0+1}$ and $\mu _k<u^t<\mu _{k+1}$, then for all $u'\in [\epsilon -u,u+\epsilon ]$ with $\epsilon >0$ sufficiently small the quotient appearing in $(\Delta '_{\Sigma _M\circ \exp })$ remains constant, i.e. in this case the crucial expression is locally constant.
- Even $k>j_0$ is valid: If $k=j_0$, then in order to have $(\Delta '_{\Sigma _M\circ \exp })$ we need $\mu _{j_0}\le u<u^t<\mu _{j_0+1}$ for all $u\ge u_0$ with $\mu _{j_0}<u<\mu _{j_0+1}$, a contradiction as $u\rightarrow \mu _{j_0+1}$.
- If $(\Delta '_{\Sigma _M\circ \exp })$ holds, then for all *u* with $\mu _{j_0}\le u_0\le u<\mu _{j_0+1}$ it follows that $u^t<\mu _{k+1}$: Otherwise, if $u^t\ge \mu _{k+1}$, then $\frac{\Sigma _M(u^t)}{\Sigma _M(u)}\ge \frac{k+1}{j_0}>\frac{k}{j_0}=\frac{\Sigma _M(u_0^t)}{\Sigma _M(u_0)}$, a contradiction.
- On the other hand, for all $u\ge u_0$ such that $\mu _k\le u^t<\mu _{k+1}$ it is allowed that $u\ge \mu _{j_0+d}$ for some $d\in \mathbb {N}_{>0}$: In this case, if $\mu _{j_0+d}\le u<\mu _{j_0+d+1}$ and still $\mu _k\le u^t<\mu _{k+1}$, then $\frac{\Sigma _M(u^t)}{\Sigma _M(u)}=\frac{k}{j_0+d}<\frac{k}{j_0}$.
- Take $u=(\mu _{k+1})^{1/t}$ and so $u>u_0$. We get that $\mu _{j_0+d}\le u<\mu _{j_0+d+1}$ for some $d\in \mathbb {N}$. Thus $\Sigma _M(u^t)=k+1$ holds (since $\mu$ is strictly increasing!) and $\Sigma _M(u)=j_0+d$. We distinguish: If $\mu _{j_0+d}<u<\mu _{j_0+d+1}$, then we can find $\epsilon >0$ sufficiently small to ensure $u-\epsilon >\mu _{j_0+d}$ and $\mu _k\le (u-\epsilon )^t<\mu _{k+1}$. In this case $\frac{\Sigma _M(u^t)}{\Sigma _M(u)}=\frac{k+1}{j_0+d}>\frac{k}{j_0+d}=\frac{\Sigma _M((u-\epsilon )^t)}{\Sigma _M(u-\epsilon )}$, a contradiction to $(\Delta '_{\Sigma _M\circ \exp })$. In particular this case happens if $d=0$ because then $\mu _{j_0}\le u_0<u$ by assumption.
- If now $\mu _{j_0+d}=u<\mu _{j_0+d+1}$ for some $d\ge 1$ and $u^t=\mu _{k+1}$, then take $\epsilon >0$ sufficiently small to ensure $u-\epsilon >\mu _{j_0+d-1}$ and $\mu _k\le (u-\epsilon )^t<\mu _{k+1}$. Both estimates are possible since the sequence $\mu$ is assumed to be strictly increasing. Thus $\frac{\Sigma _M(u^t)}{\Sigma _M(u)}=\frac{k+1}{j_0+d}\le \frac{k}{j_0+d-1}=\frac{\Sigma _M((u-\epsilon )^t)}{\Sigma _M(u-\epsilon )}$ and which verifies $(\Delta '_{\Sigma _M\circ \exp })$ in this case because this estimate is equivalent to $j_0+d-1\le k$ and this is clear since $\mu _{j_0+d}=u<u^t=\mu _{k+1}$.
- Summarizing all the information, a necessary condition to ensure $(\Delta '_{\Sigma _M\circ \exp })$ is that
  7.25 $$\begin{aligned}{} & {} \exists \;j_0\in \mathbb {N}_{>0}\;\exists \;t_0>1\;\forall \;t\ge t_0\;\exists \;k\in \mathbb {N}_{>0},\;k>j_0,\nonumber \\{} & {} \quad \exists \;d\in \mathbb {N}_{>0}:\;\;\;\mu _{k+1}=(\mu _{j_0+d})^t. \end{aligned}$$
- The equality precisely means $t=\frac{\log (\mu _{k+1})}{\log (\mu _{j_0+d})}$. However, the expression on the right-hand side can only take countable many values whereas *t* is required to belong to an uncountable set and therefore (7.25) is impossible. $\square$

We finish with the following consequence showing that in general [11, Thm. 5.1] does not provide a characterization. This should be compared with [17, Sect. 2.3, Thm. 11] where it has been shown that an N-function *F* satisfies $\Delta '$ if and only if the left-derivative of *F* has $\Delta '$.

#### Corollary 7.26

There exist N-functions *F* such that *F* satisfies the $\Delta '$-condition but $(\Delta '_{f})$ fails.

#### Proof

Consider the function(s) $\omega _s$, $s>1$. Then $\varphi _{\omega _s}(t)=t^s$ for all $t\ge 0$ and so the $\Delta '$-condition is satisfied. Since $\omega _{M^{q,n}}\sim \omega _s$ for all $q>1$ and $n>1$ such that $\frac{1}{s}+\frac{1}{n}=1$ we get that $\varphi _{\omega _{M^{q,n}}}\sim \varphi _{\omega _s}$ as well (see the proof of $(ii)\Leftrightarrow (iii)$ of Theorem 4.2). It is immediate that the $\Delta '$-condition is also preserved under $\sim$. Hence $\varphi _{\omega _{M^{q,n}}}$ and finally $F_{M^{q,n}}$ satisfy the $\Delta '$-condition. However, for any $q>1$ the corresponding sequence of quotients is clearly strictly increasing (recall that $\mu ^{q,n}_j=q^{j^n-(j-1)^n}$, $j\ge 1$) and so by Proposition 7.25 property $(\Delta '_{f_{M^{q,n}}})$ fails. $\square$
